# Conformational Analysis and NMR Data Assignment of Taraxerone Isolated From *Cnidoscolus aconitifolius* (Chaya)

**DOI:** 10.1002/mrc.70055

**Published:** 2025-10-22

**Authors:** Mónica Díaz‐Fernández, Karla Cahun‐Uicab, Viviana Roche‐Llerena, Leonardo Hernández, Geonel Rodríguez‐Gattorno, Armando Ariza‐Castolo, María A. Fernández‐Herrera

**Affiliations:** ^1^ Departamento de Física Aplicada Centro de Investigación y de Estudios Avanzados del Instituto Politécnico Nacional Merida Mexico; ^2^ Departamento de Química Centro de Investigación y de Estudios Avanzados del Instituto Politécnico Nacional Mexico City Mexico

**Keywords:** chaya, conformational analysis, hyperconjugation effect, taraxerone, γ‐effect

## Abstract

We report, for the first time, the isolation of taraxerone from an isopropanol extract of chaya ( *Cnidoscolus aconitifolius* ), a traditional plant used in Mayan culture. Although this natural product has been previously identified in other plant species, the complete assignment of its NMR data had not been accomplished until now. To achieve this, we employed 2D NMR experiments to assign the ^1^H and ^13^C chemical shifts of taraxerone. Several approaches were used to determine the spin–spin coupling constants (*J*_H,H_), including GIAO calculations, spin simulation, and dihedral angle analysis via a Karplus‐type HLA equation. Conformational analysis revealed the presence of two conformations of Ring A: a chair and a twisted boat. To better understand the behavior of these conformers, a variable‐temperature ^1^H NMR experiment was performed, in which Δ*δ*/°C values for selected protons were monitored. These changes are attributed to stereoelectronic effects, such as the Perlin effect, as evidenced by ^1^ *J*_C,H_ values obtained from HSQC nondecoupled experiments. Isotopomeric shifts (^1^Δ^12/13^C(^1^H)) were also investigated. All findings were further supported by natural bond orbital (NBO) calculations, which helped explain the observed hyperconjugative interactions.

## Graphical abstract

We report the first complete NMR assignment of taraxerone, a pentacyclic triterpenoid isolated from *Cnidoscolus aconitifolius* (chaya). Using 2D NMR, DFT, and NBO analysis, we resolved spectral ambiguities due to high‐order couplings and signal overlap. Two Ring A conformers were identified and characterized. Stereoelectronic effects such as the Perlin and γ‐*gauche* effects were quantified, highlighting the synergy between experimental and computational tools in complex structure elucidation.

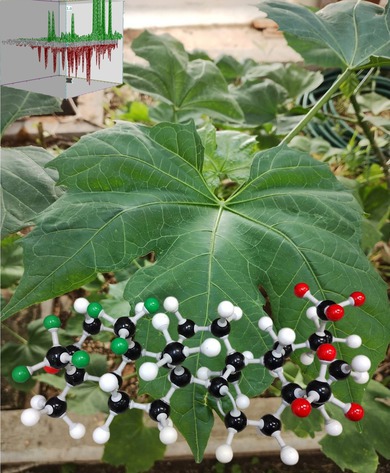

## 1 Introduction

*Cnidoscolus aconitifolius* , commonly known as chaya in Mayan culture, is used in both gastronomy and ethnobotany [1]. Although several studies have described its biological activities [2, 3], physicochemical properties [4], and partial compositional analyses of the aerial parts [5, 6], the isolation of a pure compound has not yet been achieved. Although there is a nonindexed report suggesting the presence of taraxerone in chaya, to the best of our knowledge, no peer‐reviewed study has described its isolation and spectroscopic characterization from this plant. To address this, we report here the isolation of taraxerone from the isopropanol extract of chaya leaves and, for the first time, the complete assignment of its NMR data. Taraxerone is a pentacyclic triterpenoid containing a ketone group at position 3. This natural product has previously been isolated from a range of plant species, including *Cupania dentata* [7], *Crossostephium chinense* [8], *Alnus glutinosa* [9], *Skimmia laureola* [10], *Sedum sarmentosum* [11], *Myrica cerifera* [12], and *Myrica rubra* [13].

Among the biological activities attributed to taraxerone are its antioxidant properties [11], its ability to enhance the activity of alcohol dehydrogenase and acetaldehyde dehydrogenase [14], and its sirtuin‐1‐mediated antifibrotic effects [15]. In this context, accurate structural elucidation of bioactive compounds is essential to avoid misidentification, particularly within natural product families containing structurally similar molecules that may easily be confused [16]. Nuclear magnetic resonance (NMR) spectroscopy is a powerful tool for determining molecular structure, as it provides detailed information on molecular connectivity and stereochemistry. In recent years, plant metabolomic studies have gained increasing relevance, highlighting the need for a robust NMR data library to serve as fingerprints for natural products in complex mixtures [17]. However, signal overlap is a frequent challenge in triterpene and steroid analysis, due to the chemical and magnetic similarities among protons, which lead to closely spaced signals and high‐order couplings that complicate the extraction of spin–spin coupling constants (*J*) values. To address these difficulties, several strategies have been developed. Computational approaches such as the gauge‐including atomic orbital (GIAO) method can be used to calculate chemical shifts (magnetic shielding tensors) and *J* values [18]. Additionally, coupling constants can be estimated from torsional angles [19] using Karplus‐type equations [20, 21]. There is a growing trend toward using computational methods rather than experimental synthesis or extraction when demonstrating structural features [22]. Beyond structure elucidation, NMR data assignment also provides insight into molecular geometry and dynamics in solution. For example, cyclohexane rings typically adopt chair conformations but can undergo distortions due to structural perturbations. In taraxerone, the ketone group at position 3 [23] and the two pairs of geminal methyl groups at positions 4 and 20 promote 1,3‐diaxial interactions with adjacent methyl groups, leading to deviations from ideal bond angles.

**FIGURE 1:**
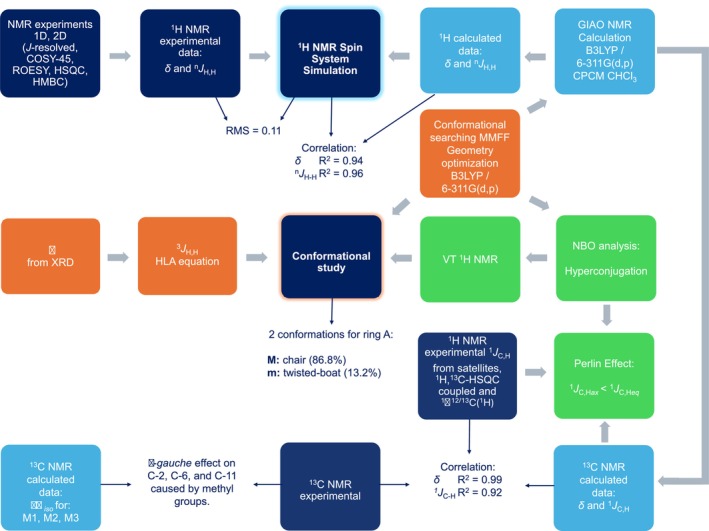
Workflow for NMR data assignment and conformational study of taraxerone.

In this work, we investigated the conformational preferences of taraxerone and achieved the complete ^1^H and ^13^C NMR assignment through a combined approach involving 2D NMR techniques, spin simulation, and computational methods, with the aim of providing a reliable structural fingerprint and gaining deeper insight into its stereoelectronic behavior in solution. The strategies employed in this study are outlined in the subsequent workflow (Figure 1).

## 2 Materials and Methods

### 2.1 General Experiment Procedures

The NMR spectra of taraxerone were determined on a JEOL ECA 500 (^1^H, 500.15992 MHz; ^13^C, 125.7653 MHz) spectrometer equipped with an 11.74736‐T magnet and a 5‐mm indirect‐detection probe with *z* gradients and controlled with Delta NMR Processing and Control Software [24]. All experiments were carried out using a solution in CDCl_3_. The ^1^H spectrum was acquired with an offset of 4 ppm, a sweep of 5.004 kHz, 16,384 data points, a digital resolution of 0.30542 Hz, an equivalent 45° pulse duration, and 32 scans, with a recycle delay of 1 s. The low‐temperature spectra were recorded with the same conditions. The ^13^C satellites were determined to use the same parameters with 16,384 scans.

The ^13^C{^1^H} spectrum was acquired with an offset of 100 ppm, a sweep of 31.44654 kHz, 32,768 data points, a digital resolution of 0.95967 Hz and 7000 scans, a pulse width of 30°, and a recycle delay of 0.1 s, using the standard JEOL pulse sequence single‐pulse decoupling with NOE. Similar conditions were used for the INEPT no decoupling spectrum.

COSY‐45 spectra were obtained with the standard pulse sequence [25] using a 2048 × 512 data point matrix and a 2.57294 × 2.57294‐kHz frequency matrix. The recycle delay was 1.5 s, and a total of four scans were performed. Fourier transformations were carried out for F1 (zerofill of 4) and F2 using a sine function in the absolute‐value mode.

2D pfg‐HSQC zz‐filter edit‐phase spectrum [26] using a 1024 × 256 data point matrix and a 2.5015 × 8.80592‐kHz frequency matrix. Using a *J* = 130 Hz. The recycle delay was 1.5 s, and eight scans were performed. Fourier transformations were carried out using an exponential function of 3 Hz for F1 (zerofill of 2) and F2.

2D pfg‐HSQC with BIRD pulse without ^1^H decoupling spectrum [27] using a 4096 × 128 data point matrix and a 2.015 × 7.546‐kHz frequency matrix. Using a *J* = 130 Hz. The recycle delay was 1 s, and a total of 56 scans were performed. Fourier transformations were carried out using an exponential function of 29 Hz for F1 (zerofill of 2) and an exponential function of 0.5 Hz for F2.

2D pfg‐HMBC pulse sequence [28] was recorded using a recycle delay of 1.5 s, F2 offset of 1.5 ppm, and F1 offset of 115 ppm, using a 2048 × 256 data point matrix and a 2.5015 × 27.686‐kHz frequency matrix. Using ^1^ *J*_CH_ = 140 Hz, long‐range *J* of 4 Hz. Processing parameters: zerofill of 1 in the F1 dimension and an exponential window function.

2D t‐ROESY spectra were obtained with the standard pulse sequence [25] using a 1024 × 256 data point matrix and a 3.14209 × 3.14209‐kHz frequency matrix. The mix time was 1 s, the recycle delay was 1.5 s, and a total of four scans were performed.

2D *J*‐resolved experiment was performed using the HOMO‐*J*‐resolved pulse sequence [25], acquisition time 0.63652 s, recycle delay 1 s, offset 3.1529 ppm, using a 2048 × 128 data point matrix, and a 3.2175 × 50‐Hz frequency matrix.

X‐ray crystallographic data were collected on a Bruker D8 VENTURE diffractometer with a microfocus sealed tube using a multilayer mirror as a monochromator and a PHOTON 100 detector. The diffractometer used Mo *Kα* radiation (*λ* = 71.073 pm). Low‐resolution mass spectra were determined on an Agilent Technologies 6120 Quadrupole LC/MS coupled to HPLC 1200 Series. IR spectra were measured using an ATR interface on an Agilent Cary 630 FTIR spectrometer (4000–600 cm^−1^). Optical rotations were measured at 25°C in an Anton Paar MCP‐500 polarimeter. The melting point was determined on an Electrothermal IA9000 apparatus and is uncorrected. Column chromatography was performed in a Teledyne Isco Combiflash apparatus and analytical thin‐layer chromatography (TLC) on aluminum plates precoated with Silica Gel 60F‐254.0.

### 2.2 Plant Material

*Cnidoscolus aconitifolius* (Mill.) I. M. Johnst was collected in Dzitya, Mérida, Yucatán, Mexico, at 10 masl (21°03′25.2″ N 89°39′56.8″ W) on March 6, 2025. It was identified by Biol. J. L. Tapia‐Muñoz at the Herbarium “U najil tikin xiw” of the Centro de Investigación Científica de Yucatán (CICY) and deposited with voucher number 001. The chaya leaves were treated as established in a preceding study [4]; then, 34 g of the dried leaf powder was extracted with 0.5 L of 2‐propanol at room temperature. The mixture was stirred at 300 rpm for 48 h. Thereafter, the extract was filtered through Whatman paper with a 2.7‐μm pore size. The resulting solution was concentrated and subsequently subjected to column chromatography with a gradient of hexane/ethyl acetate from 10:0 to 9:1. The compound was obtained as 0.1352 g (0.4% yield) of colorless needle crystals with a melting point of 244°C, as confirmed by differential scanning calorimetry (DSC).

Taraxerone: Colorless crystals, m.p. 244°C; [α]_589_ + 22.5 (*c*); IR ${\bar{\nu }}_{\max}$ 2926 (C–H asymmetric stretching), 2853 (C–H symmetric stretching), 1707 (C=O stretching), 1448 (C–H bending), 1375 (C–O stretching) cm^−1^; NMR ^1^H, 500.160 MHz; ^13^C, 125.765 MHz, CDCl_3_; see Table 2; ESI‐MS: *m/z* found [M + H]^+^ 425.4 and [^12^C_29_^13^CH_49_O]^+^ 426.4.

### 2.3 Computational Studies

Monte Carlo search of taraxerone was performed using MMFF94 as implemented in Spartan'24 [29]. The single‐point energy of each conformer was performed at the HF 6‐31G* level of theory. The two most stable conformers were reoptimized at the B3LYP/6‐311G(d,p) level in Gaussian'09 [30]. The optimized structures were compared to those obtained with the functional ωB97XD/def2‐TZVP, using chloroform as solvent, and there were no significant changes. The coupling constants and shielding tensors were obtained using the GIAO approach. These calculations were carried out at the B3LYP/6‐311G(d,p) level with chloroform as solvent using conductor‐like polarizable continuum model (CPCM) [31] as the implicit solvent model. The stationary points were characterized by performing harmonic vibrational frequency analysis, and their relative energies were used to calculate the Boltzmann distribution.

## 3 Results and Discussion

**FIGURE 2:**
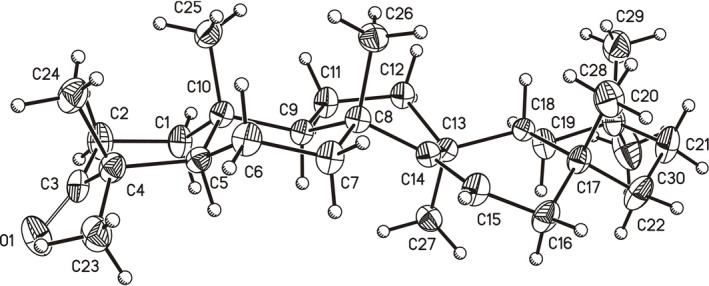
ORTEP diagram of taraxerone, with displacement ellipsoids shown at the 30% probability level. This structure was determined and refined in the present work and is consistent with previously reported data [10].

Taraxerone was isolated from the isopropanol extract of chaya leaves, following the extraction method previously reported by our research group [4]. After purification, the triterpenoid was subjected to comprehensive spectroscopic analysis by NMR. Structural elucidation was further supported by mass spectrometry (MS), infrared (IR) spectroscopy, optical rotation and melting point determination. These results were corroborated by single‐crystal X‐ray diffraction (Figure 2).

**FIGURE 3:**
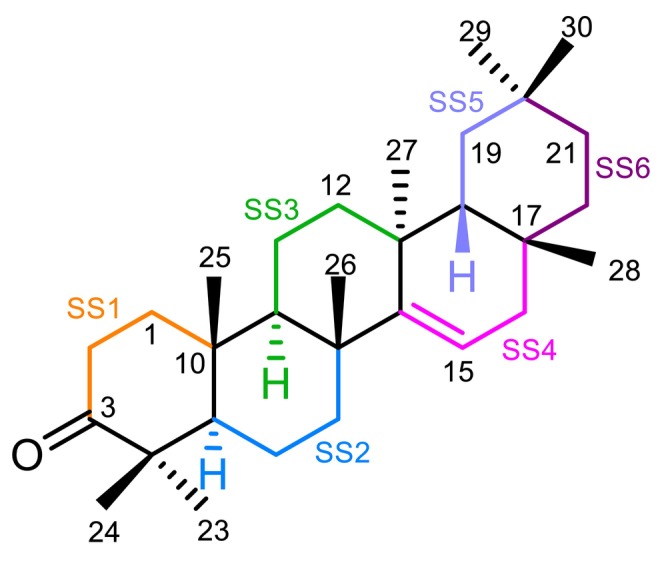
Molecular structure of taraxerone, with spin systems (SSs) highlighted in different colors.

**FIGURE 4:**
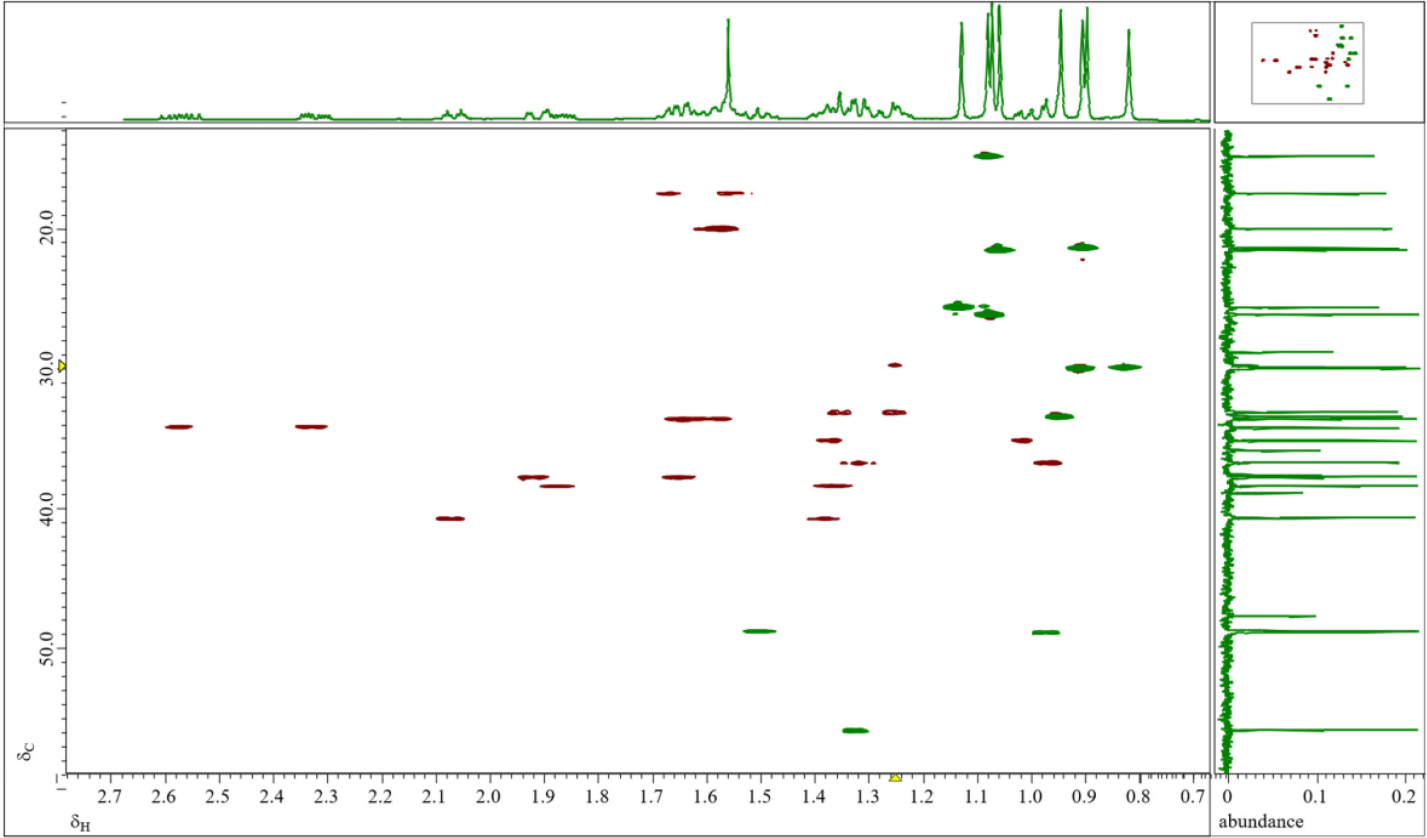
Top: ^1^H NMR; vertical right: ^13^C NMR; 2D pfg‐HSQC zz‐filter edit‐phase spectrum: positive phase in green (CH and CH_3_), negative phase in red (CH_2_).

The ^1^H NMR analysis was facilitated by dividing the proton resonances into six spin systems (SSs), based on the transfer of magnetization between protons via *J* couplings, which are interrupted by quaternary carbon atoms (Figure 3). In the ^1^H NMR spectrum, the most distinguishable resonances were associated with SS4, that is, the vinylic (H‐15) and allylic (H‐16_*ax*_) protons, as well as the protons in Ring A (SS1, except for H‐1_*ax*_). The ketone group at position 3 of Ring A produces a deshielding effect, resulting in a noticeable chemical shift compared to other protons in the molecule. Taking advantage of these well‐resolved signals, coupling constants were extracted, and correlations from HSQC (Figure 4) and HMBC experiments were used to assign carbon resonances through one‐bond (C‐1, C‐2, C‐15, and C‐16) and multiple bond (C‐3, C‐4, C‐9, C‐10, C‐13, C‐17, and C‐18) interactions.

**FIGURE 5:**
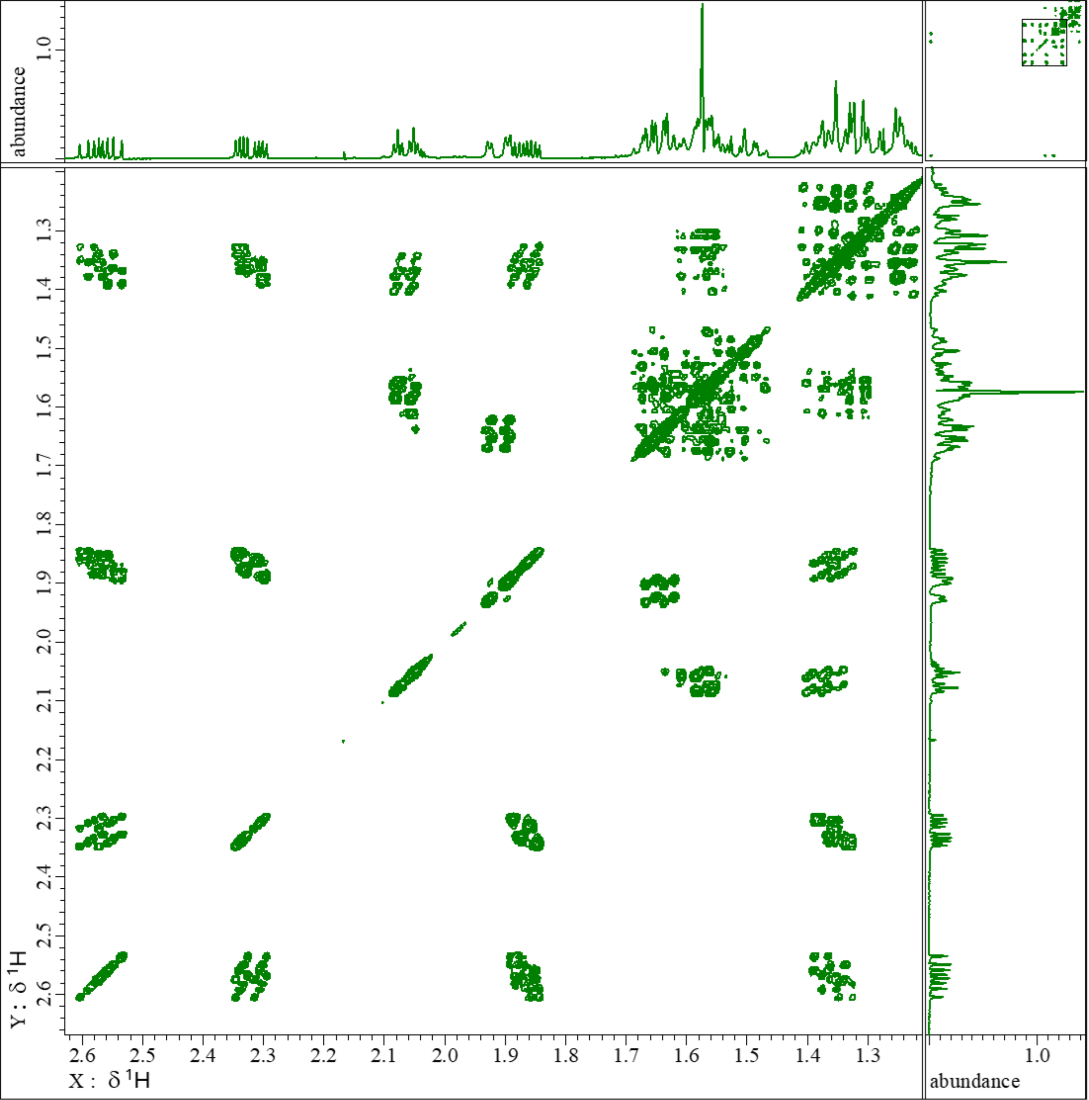
Top and vertical right: ^*1*^H NMR; 2D COSY‐45 positive tilt indicates relative sign of passive ^*n*^ *J*_*x*_/^*n*^ *J*_*y*_ > 0, negative tilt indicates relative sign of passive ^*n*^ *J*_*x*_/^*n*^ *J*_*y*_ < 0.

The COSY‐45 experiment provided valuable information by allowing the distinction of geminal and vicinal proton couplings based on the sign of the cross‐peaks, which tilt positively or negatively, respectively [32]. Notable examples of clearly resolved geminal couplings included protons at positions 1, 2, 16, and 19. In more complex regions, signal overlap made the interpretation of cross‐peak signs more difficult (Figure 5).

Additionally, a ROESY experiment (see the Supporting Information) was performed to confirm SS connectivity (Figure 2). However, due to extensive signal overlap, the certainty of some assignments was limited. This experiment was also used to help differentiate between α‐ and β‐oriented protons. For this purpose, experimental *J* values and torsional angle analysis were jointly evaluated.

**FIGURE 6:**
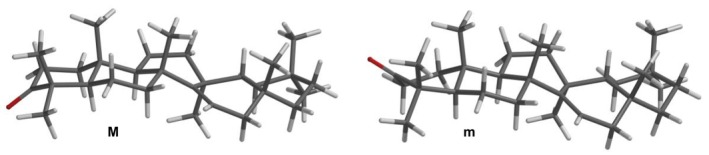
The most stable density functional theory (DFT) B3LYP/6‐311G(d,p) majority (**M**) and minority (**m**) conformers of taraxerone.

In parallel, a conformational analysis was performed using the Merck molecular force field (MMFF) methods, followed by single‐point and geometry optimization calculations. This analysis identified two stable conformers that significantly contribute to the Boltzmann distribution: conformer **M** (86.8%) and conformer **m** (13.2%) (Table 1 and Figure 6). The primary structural difference between these conformers lies in the geometry of Ring A. The chair conformation was found to be the most stable, while the twisted‐boat conformation corresponded to a higher energy state. In the solid state, the chair form predominates. It is reasonable to expect that both conformers coexist in solution, and their presence can be confirmed by NMR spectroscopy, as they influence dihedral angles and, consequently, the spin–spin coupling constants observed in liquid‐phase spectra.

**TABLE 1:** Thermochemical parameters of the most stable DFT B3LYP/6‐311G(d,p) conformers of taraxerone.

Conf.	Δ*E*_MMFF94_ (kJ/mol)	%^a^	Δ*E*_6‐31G*_ (kJ/mol)	%^a^	B3LYP	
					Δ*E*_6‐311G(d,p)_ (kJ/mol)	%^a^
**M**	0.00	76.8	0.00	80.8	0.00	86.8
**m**	3.05	23.2	3.56	19.2	4.64	13.2

^a^ Calculated according to Δ*E*≈−*RT*ln*K*.

**FIGURE 7:**
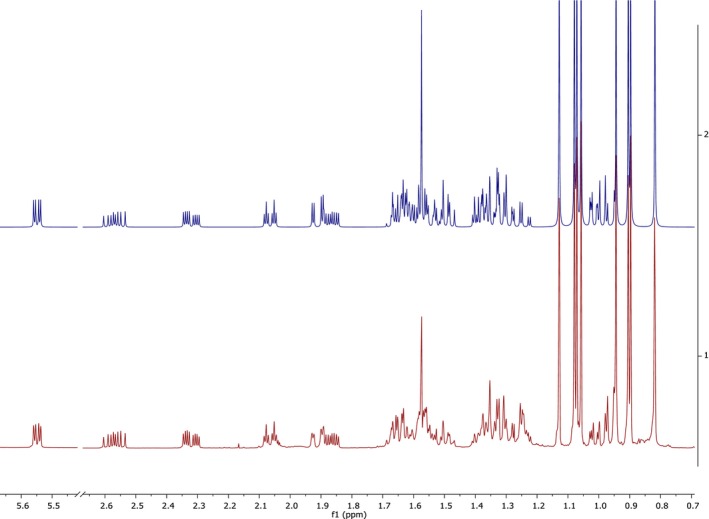
Bottom: experimental ^1^H NMR @ 500.15992 MHz in CDCl_3_; top: simulated spectrum.

Following the identification of the most stable conformers, GIAO NMR calculations with the implementation of CPCM for chloroform were performed. From these, both the coupling constants and their signs were obtained. These computational data, together with the experimental *δ* and *J* values, were used to perform spin simulations using SpinWorks [33] and MestReNova [34]. As previously noted, the molecule was divided into six SSs (see Figure 3), as established in the ROESY experiment. The simulated values were adjusted iteratively until an adequate match with the experimental ^1^H NMR spectrum was achieved (Table 4). Initially, a visual comparison was carried out, followed by root mean square (RMS) error analysis, which yielded a value of 0.11, indicating good agreement between the simulated and experimental data (Figure 7). The iterative ^1^H full spin analysis (HiFSA) is increasingly employed due to its effectiveness in deconvoluting overlapped NMR signals [35], as shown here in the full assignment of taraxerone. The combination of different NMR methodologies, 2D correlations, spin simulation, and computational calculations enabled the complete and confident assignment of both ^1^H and ^13^C NMR data.

In consideration of the ^13^C NMR presented data (Table 2), a notable γ‐*gauche* effect was observed on carbon atoms 2, 6, and 11, caused by Me‐23, Me‐24, and Me‐25 for C‐2 (*δ =* 34.1); Me‐23, Me‐24, Me‐25, and Me‐26 for C‐6 (*δ =* 19.9); and Me‐25, Me‐26, and Me‐27 for C‐11 (*δ* = 17.5). This effect resulted in the shielding of the carbon nuclei implicated [36]. To verify the influence of methyl groups on the γ‐effect, three different GIAO (B3LYP/6‐311G(d,p)) calculations were performed using the optimized geometry of **M**. In the first calculation, all Me were deleted (**M1**). In the second calculation, only Me in *equatorial* positions were conserved (**M2**). In the third calculation, Me were kept in *axial* positions (**M3**). The data obtained from calculations revealed that the methyl groups in *axial* positions exert the most significant effect on their respective γ‐carbons (Table 3). In particular, the calculation outcomes indicated that the γ‐*gauche* effect induced by the four methyl groups on C‐6 is greater than the observed effect for C‐11. However, this is not reflected in the experimental *δ* (Table 2) due to the γ‐*anti* effect of the carbonyl group (*ϕ*_C‐2–C‐4–C‐5–C‐6_ = 179.6°). Conversely, the γ‐*anti* effect exhibited by *equatorial* Me‐24 on C‐2 is negligible in comparison to the γ‐*gauche* effect caused by Me‐23 and Me‐25.

**TABLE 2:** Assignment of NMR data and selected correlations observed in 2D experiments.

Position	*δ* ^1^H	*δ* ^13^C	HMBC	COSY	ROESY
1_*ax*_	1.372	38.3	C‐4, C‐9	H‐1_*eq*_, H‐2_*ax*_, H‐2_*eq*_	H‐1_*eq*_, H‐5_*ax*_, H‐9_*ax*_
1_*eq*_	1.867		C‐2, C‐10	H‐1_*ax*_, H‐2_*ax*_, H‐2_*eq*_	H‐2_*ax*_, H‐2_*eq*_, H‐11_eq*_, Me‐25
2_*ax*_	2.568	34.1	C‐2, C‐3	H‐1_*ax*_, H‐1_*eq*_, H‐2_*eq*_	H‐2_*eq*_, Me‐25, Me‐23
2_*eq*_	2.321		C‐10	H‐1_*ax*_, H‐1_*eq*_, H‐2_*ax*_	H‐2_*ax*_
3		217.7			
4		47.6			
5_*ax*_	1.315	55.7	C‐6, C‐9, C‐10, C‐23, C‐24		H‐1_*ax*_, H‐5_*ax*_, H‐9_*ax*_, Me‐24
*6*_*ax*_	1.520	19.9	C‐7, C‐8		
*6*_*eq*_	1.619		C‐5, C‐7, C‐10	H‐6_*ax*_, H‐7_*eq*_	
7_*ax*_	1.360	40.6	C‐8	H‐6_*ax*_, H‐7_*eq*_	H‐1_*ax*_, H‐6_*ax*_, H‐7_*eq*_, Me‐25
7_*eq*_	2.065		C‐4, C‐6, C‐8, C‐26	H‐6_*ax*_, H‐6_*eq*_, H‐2_*ax*_	H‐6_*ax*_, H‐6_*eq*_, Me‐26
8		38.8			
9_*ax*_	1.488	48.7	C‐11		H‐5_*ax*_, H‐7_*ax*_, Me‐23, Me‐25, Me‐27
10		37.7			
11_ax*_	1.570	17.5	C‐9		
11_eq*_	1.643		C‐9, C‐12, C‐13, Me‐26		
12_*ax*_	1.316	36.6	C‐11, C‐13		Me‐28
12_*eq*_	1.003				H‐11_ax*_, H‐11_eq*_, Me‐25
13		28.8			
14		157.6			
15	5.548	117.2	C‐8, C‐16, C‐17	H‐16_*ax*_, H‐16_*eq*_	H‐7_*eq*_, H‐7_*ax*_, H‐16_*eq*_
16_*ax*_	1.910	37.7	C‐13, C‐15, C‐18	H‐15, H‐16_*eq*_	H‐19_*ax*_
16_*eq*_	1.646		C‐15, C‐17	H‐15, H‐16_*ax*_	H‐15, H‐16_*ax*_, Me‐28
17		35.8			
18_*ax*_	0.962	48.7			
19_*ax*_	1.326	35.1	C‐21, C‐20	H‐18_*ax*_, H‐19_*eq*_	
19_*eq*_	1.013		C‐18, C‐20, C‐21, C‐29, C‐30	H‐19_*ax*_	Me‐29
20		37.5			
21_*ax*_	1.254	33.0	C‐19, C‐22	H‐21_*eq*_	H‐18_*ax*_, Me‐28
21_*eq*_	1.387		C‐19, C‐20	H‐21_*ax*_	
22_*ax*_	1.560	33.5	C‐20		Me‐29
22_*eq*_	1.647		C‐18, C‐20		
23	1.071	26.1	C‐1, C‐3, C‐4, C‐5, C‐9, C‐24		H‐2_*ax*_, H‐6_*ax*_
24	1.057	21.5	C‐3, C‐4, C‐5		H‐1_*ax*_, H‐5_*ax*_
25	1.079	14.8	C‐9, C‐5		H‐6_*ax*_, Me‐23, Me‐26
26	1.128	25.6	C‐7, C‐8, C‐9, C‐14		H‐7_*eq*_, Me‐25
27	0.944	33.3	C‐13		H‐7_*ax*_, H‐11_*ax*_, H‐19_*ax*_
28	0.818	29.8	C‐16, C‐17, C‐18, C‐22		H‐16_*eq*_, H‐21_*ax*_, Me‐26, Me‐30
29	0.905	29.9	C‐18, C‐19, C‐20, C‐21, C‐30		H‐19_*ax*_, H‐19_*eq*_, H‐22_*ax*_,
30	0.897	21.3	C‐18, C‐19, C‐20, C‐21, C‐29		Me‐28

**TABLE 3:** GIAO‐calculated isotropic shielding tensor differences (Δ*σ*) resulting from the γ‐effect on C‐2, C‐6, and C‐11.

Carbon	Δ*σ* **M1**	Δ*σ* **M2**	Δ*σ* **M3**	*ϕ* (°)
2	−6.7	0.2^a^	−6.3	Me‐23–C‐4–C‐3–C‐2 = 75.7 Me‐24–C‐4–C‐3–C‐2 = −167.4^a^ Me‐25–C‐10–C‐1–C‐2 = −69.6
6	−11.3	−3.6	−8.4	Me‐23–C‐4–C‐5–C‐6 = 58.8 Me‐24–C‐4–C‐5–C‐6 = −62.6 Me‐25–C‐10–C‐5–C‐6 = −64.3 Me‐26–C‐8–C‐7–C‐6 = 76.0
11	−6.4		−6.3	Me‐25–C‐10–C‐9–C‐11 = −63.2 Me‐26–C‐8–C‐9–C‐11 = 62.1 Me‐27–C‐13–C‐12–C‐11 = 65.1

^a^ γ‐*anti* effect.

**FIGURE 8:**
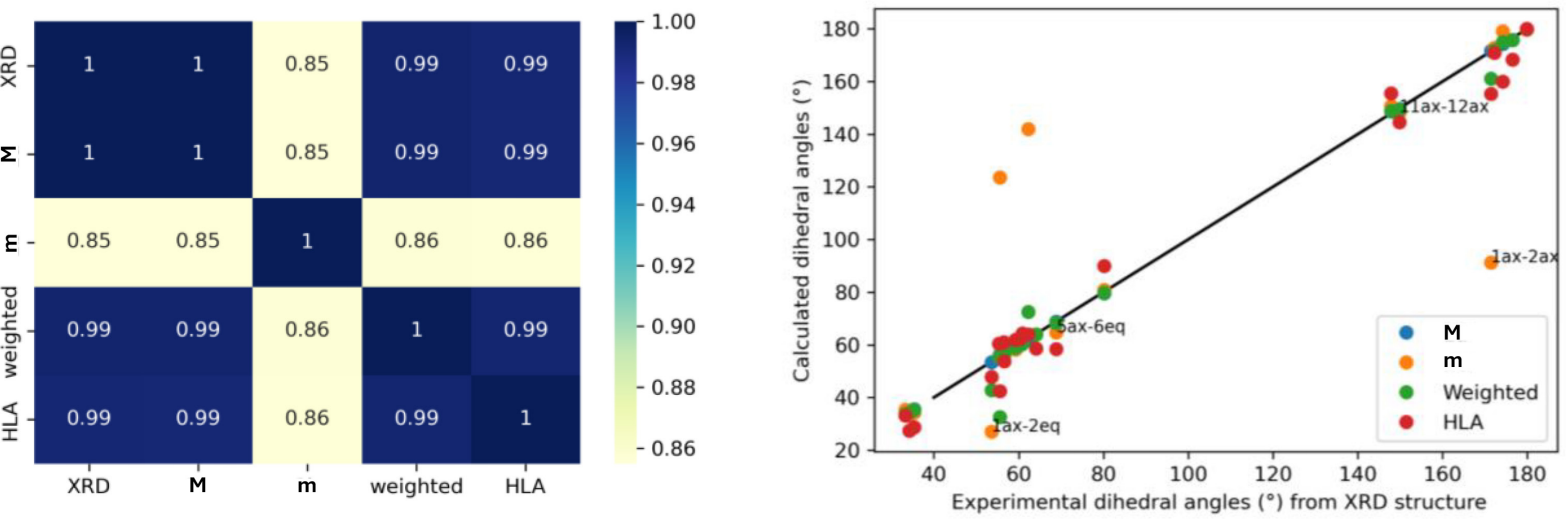
Heatmap (left) and scatter plot with identity line (right) showing the correlation between the torsional angle datasets for taraxerone (see text).

The focus is again redirected to ^1^H NMR data. An approach to validate the experimentally obtained *J* values involves measuring the torsional angles for each conformer and calculating their weighted average. These averaged angles were then evaluated using the Haasnoot–de Leeuw–Altona (HLA) [37] equation within MestReJ software. This method was chosen because the HLA equation accounts for the electronegativity of substituents, providing a more realistic approximation than the traditional Karplus equation. In a complementary analysis, the experimental *J* values (Table 4) were used to calculate the corresponding dihedral angles via the same HLA equation. The resulting four sets of torsional angles, those derived from conformers, experimental *J* values, and X‐ray crystallography (XRD), were compared. This comparison was visualized using two graphical tools: a heatmap and a scatter plot with an identity line (Figure 8). The heatmap facilitated a global comparison among the datasets by depicting the strength of correlations through a color gradient. Notably, a strong correlation was observed between the **M** conformer and the XRD‐derived structure. Additionally, high consistency was found between the HLA‐derived angles and the weighted conformers, indicating the contribution of the twisted‐boat (**m**) conformation of Ring A to the variation in some dihedral angles. Nonetheless, the remaining angles showed strong alignment across all models.

**TABLE 4:** Spin–spin coupling constants (^*n*^ *J*_H,H_, Hz) for the systems SS1–SS6.

SS1	1_*ax*_	1_*eq*_	2_*ax*_		SS4	15	16_*ax*_	
1_*eq*_	−13.25				16_*ax*_	+3.15		
2_*ax*_	+11.9	+7.1			16_*eq*_	+8.2	−14.82	
2_*eq*_	+6.32	+3.3	−15.9					

SS2	5_*ax*_	6_*ax*_	6_*eq*_	7_*ax*_	SS5	18_*ax*_	19_*ax*_	
6_*ax*_	+11.21				19_*ax*_	+14.5		
6_*eq*_	+3.95	−10.39			19_*eq*_	+3.62	−12.53	
7_*ax*_	+0.11	+13.77	+4.8					
7_*eq*_	−0.1	+3.3	+3.3	−12.88				

SS3	9_*ax*_	11_*ax*_	11_*eq*_	12_*ax*_	SS6	21_*ax*_	21_*eq*_	22_*ax*_
11_*ax*_	+10.7				21_*eq*_	−13.37		
11_*eq*_	+7.9	−14.9			22_*ax*_	+13.5	+2.85	
12_*ax*_	−0.4	+10	+13.2		22_*eq*_	+3.2	+3.52	−15.94
12_*eq*_	−0.2	+0.3	+9.7	−13.9				

These findings support the reliability of both the conformational analysis and the Boltzmann population estimates derived from theoretical calculations. The use of complementary plots, particularly the scatter plot, enabled the identification of specific dihedral angles that deviated from the general trend, thereby highlighting regions of conformational flexibility and reinforcing the validity of the integrated NMR and computational approach.

**FIGURE 9:**
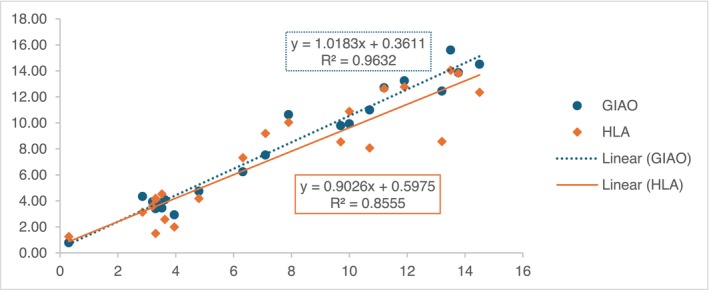
Correlation between vicinal *J*_H,H_ experimental, calculated using GIAO B3LYP/6‐31G(d,p) and obtained from HLA equation.

Additionally, the values of weighted ^3^ *J*_H,H_ were compared with the experimental ones and contrasted with the values obtained from the HLA equation. As indicated by the coefficient of determination (*R* ^2^), the GIAO‐calculated coupling constants show enhanced correlation (Figure 9). While the results obtained with the HLA equation supported the conformational analysis, the similarity with the experimental *J* was found to be deficient due to the oversimplification of the stereoelectronic contribution of the substituents nearby the protons implied [37].

**FIGURE 10:**
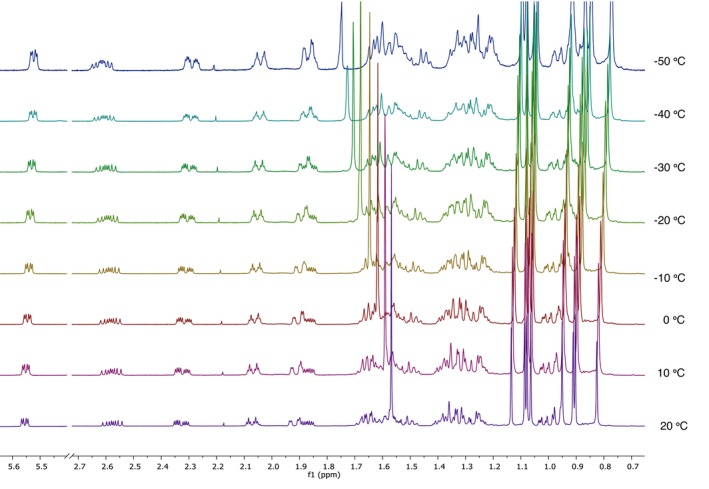
^1^H NMR spectra at temperatures ranging from 20°C to −50°C @ 500.15992 MHz/CDCl_3_.

A variable‐temperature (VT) ^1^H NMR experiment was performed to investigate the conformational dynamics of Ring A and to determine whether the chair conformation becomes more predominant at lower temperatures (Figure 10). As the temperature decreased, the splitting patterns remained consistent, although small variations in chemical shift per unit temperature (Δ*δ*/°C) were observed. Notably, the H‐2_*ax*_ exhibited a Δ*δ*/°C = −5.3 × 10^−4^ indicating a shift toward higher frequencies, opposite to the behavior observed for most other protons (Table 5).

**TABLE 5:** Differences in chemical shifts per unit of temperature (Δ*δ*/°C), one‐bond carbon–proton coupling constant (^*1*^ *J*_C,H_), isotopomeric chemical shifts (^1^Δ^12/*13*^C(^*1*^H)) of selected resonances observed in ^*1*^H NMR, and the *s* bond character of each hydrogen with the corresponding carbon.

Position	VT Δ*δ*/°C	^1^ *J*_C,H_ ^a^	^1^ *J*_C,H_ (calc)^b^	Δ*J* ^a^	^1^Δ^12/13^C(^1^H)^c^	*s* ^d^
H‐1_*ax*_		126.93	130.91	−1.07		0.2373
H‐1_*eq*_	0.13	125.86	134.56		4.8	0.2448
H‐2_*ax*_	−0.53	124.96	128.53	5.91	−36.2	0.2372
H‐2_*eq*_	0.56	130.87	136.55		12	0.2481
H‐5_*ax*_		121.52	126.56			0.2173
H‐6_*ax*_		121.03	128.26	8.36		0.2401
H‐6_*eq*_		129.39	131.37			0.2428
H‐7_*ax*_		123.98	130.70	2.95		0.2358
H‐7_*eq*_	0.44	126.93	131.46		5.5	0.2408
H‐9_*ax*_		121.52	126.59			0.2186
H‐15	0.47	155.1	161.18		2.4	0.3035
H‐16_*ax*_	0.68	130.1	130.72	< DR	8.2	0.2368
H‐16_*eq*_		130.1	130.96			0.2409
H‐22_*ax*_		125.46	128.57	1.96		0.2375
H‐22_*eq*_		127.42	130.60			0.2414
Me‐23	0.44	126.93	132.13			0.2462
Me‐24	0.35	127.42	131.70			0.2463
Me‐25	0.11	124.47	129.33			0.2452
Me‐26	0.58	125.46	130.22			0.2456
Me‐27	0.54	123.98	130.57			0.2462
Me‐28	0.78	124.47	129.25			0.2443
Me‐29	0.65	123.98	129.03			0.2428
Me‐30	0.85	125.95	128.84			0.2428

Abbreviation: DR: digital resolution.

^a^ Obtained from HSQC nondecoupled experiment.

^b^ Calculated using GIAO at B3LYP/6‐311G(d,p) theory level.

^c^ Given in ppb.

^d^ Calculated using NBO at B3LYP/3‐21G theory level for **M**.

A plausible explanation for this distinct behavior is that at lower temperatures, the **M** conformer becomes more populated. In this conformer, the *σ*_C‐2,H‐2*ax*_ → π*_C=O_ hyperconjugation is favored, resulting in a weakened bond and a smaller one‐bond coupling constant (^1^ *J*_C‐2,H‐2*ax*_ = 124.96 Hz), compared to that of *J*_C‐2,H‐2*eq*_ (130.87 Hz), yielding a Δ^1^ *J*_C,H_ = 5.91. (Table 5). This stereoelectronic phenomenon is consistent with the Perlin effect [38, 39], where the coupling constant is modulated by the *s* character of the hybrid orbital involved, as supported by semiempirical relationships [40]. In contrast, H‐2_*eq*_ is orthogonal to the *π* orbital of the adjacent carbonyl at C‐3 and does not participate significantly in hyperconjugation.

To support this hypothesis, a natural bond orbital (NBO) analysis was conducted (Table 6). The analysis confirmed a significant *σ*_C‐2,H‐2*ax*_ → π*_C‐3=O_, interaction with an associated stabilization energy of 19.92 kJ/mol. Meanwhile, the interaction between the *σ* orbital C‐2–H‐2_*eq*_ and the carbonyl π* orbital was negligible, with an energy below the threshold (2.09 kJ/mol) typically considered relevant in Fock matrix analysis.

**TABLE 6:** Selected data from second‐order perturbation theory analysis of the Fock matrix in the NBO basis set (B3LYP/3‐21G).

Donor	Acceptor	Energy (kJ/mol)
*σ* C‐2–H‐2_*ax*_	σ* C‐3–O	5.40
	π* C‐3–O	19.92
*σ* C‐2–H‐2_*eq*_	σ* C‐3–O	3.56
*π* C‐4–O	σ* C‐4–C‐23	7.49
*σ* Me‐25	σ* C‐10–C‐9	9.41
	σ* C‐24–H‐24	3.39
	σ* C‐5–C‐10	10.92
	σ* C‐26–H‐26	2.85
*σ* Me‐23	σ* C‐3–C‐4	10.17
	π* C‐3–O	2.09
	σ* C‐4–C‐5	13.18
	σ* C‐4–C‐24	8.49
*σ* Me‐30	σ* C‐19–C‐20	11.25
	σ* C‐20–C‐29	10.46
	σ* C‐20–C‐21	10.75
*σ* Me‐28	σ* C‐17–C‐22	10.17
	σ* C‐16–C‐17	10.08
	σ* C‐17–C‐18	11.00

The most pronounced Δ*δ*/°C effects were observed for the methyl groups at positions 28 and 30, both located on Ring E (Table 5). To understand this behavior, we compared them with other methyl groups that showed minimal temperature‐dependent shifts. For instance, Me‐25, located at the A/B ring junction, is involved in two 1,3‐diaxial interactions, with Me‐23 (on Ring A) and Me‐26 (at the B/C junction). These stereoelectronic interactions increase the rigidity and electron density around Me‐25, making it less responsive to external perturbations. Supporting this, NBO analysis revealed strong orbital interactions between these methyl groups and adjacent carbons (Table 6), while torsional angles near 180° (*ϕ*_C‐25–C‐10–C‐23–C‐4_ = 175.3° and *ϕ*_C‐25–C‐10–C‐26–C‐8_ = 178.9°) indicate effective *antiperiplanar* overlap. Conversely, Me‐28 and Me‐30 are more flexible due to the absence of such stabilizing interactions. Their greater mobility can be attributed to both the spatial separation between them (43 pm) and a suboptimal torsional angle for orbital overlap (*ϕ*_C‐30–C‐20–C‐28–C‐17_ = 151.5°), which reduces conformational constraint.

**FIGURE 11:**
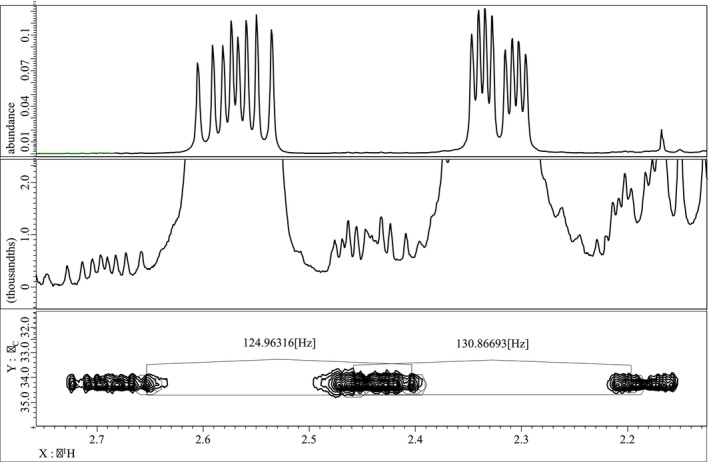
Top: ^1^H NMR spectrum showing the H‐2_*ax*_ and H‐2_*eq*_ region; middle: ^*13*^C satellite signals; bottom: 2D HSQC with BIRD pulse sequence.

To directly measure the ^1^ *J*_C,H_ values for the protons listed in Table 5, an INEPT nondecoupled experiment was conducted. However, due to the proximity of carbon chemical shifts and complex splitting patterns, reliable data could not be extracted. To address this, a nondecoupled HSQC experiment (Figure 11) was conducted instead, which provided sufficient resolution by leveraging the ^13^C satellite correlations from ^1^H signals. In parallel, an accumulated ^1^H spectrum was recorded to observe satellite peaks arising from naturally abundant ^13^C nuclei, allowing for the determination of isotopomeric chemical shifts (^1^Δ^12/13^C(^1^H)) (Table 5).

We then examined whether a correlation existed between the hyperconjugative effect and the *s* character of the bonds, as a potential explanation for the observed Δ*δ*/°C values at low temperatures. However, the hypothesis was not fully supported, as isotopomeric shifts are influenced by multiple factors beyond just hybridization [41]. Interestingly, a coincidence was observed between the negative sign of Δ*δ*/°C and ^1^Δ^12/13^C(^1^H), suggesting a deviation from the general trend (Table 6). This could be explained by electron donation from *σ*_C‐2,H‐2*ax*_ into the carbonyl π* orbital, while most other C–H bonds act as electron acceptors in their respective vicinal interactions. An inverse Perlin effect was also noted for H‐1_*ax*_ and H‐1_*eq*_.

## 4 Conclusions

Taraxerone was isolated from isopropanol extracts of chaya leaves, and its structural characterization was revisited through a comprehensive analytical approach. The dihedral angles analysis was then used with the HLA equation to estimate the vicinal *J*. Following an iterative process, a good agreement between the experimental and simulated ^1^H NMR spectra was achieved, and a strong correlation between the data obtained by these methods was confirmed. In addition, the ^13^C resonances were assigned, and the γ‐*gauche* effect was observed on C‐2, C‐6, and C‐11, induced by methyl groups at three‐bond interactions. Computational analysis determined that the chair conformation of Ring A is the most stable, which was later corroborated by X‐ray diffraction data. In the VT ^1^H NMR experiment, as the temperature decreased, the chemical shift of H‐2_*ax*_ moved in the opposite direction compared to most other protons. This was attributed to a hyperconjugative interaction between the bonding *σ* orbital of C‐2–H‐2_*ax*_ and the antibonding *π* orbital of the carbonyl group. This interaction is enabled by the unique *antiperiplanar* alignment of H‐2_*ax*_ with respect to the carbonyl, as confirmed by NBO calculations and supported by the Perlin effect Δ*J*. In most cases, the ^1^ *J*_C,H*ax*_ values were lower than the corresponding ^1^ *J*_C,H*eq*_ values. The isotopomeric chemical shifts were determined by the ^13^C satellites observed in the ^1^H spectrum outside of the overlapping region. However, until now, we cannot provide a reasonable explanation for the relationship between VT Δ*δ*/°C and ^1^Δ^12/13^C(^1^H). The major effect VT Δ*δ*/°C toward low frequencies was observed for methyl groups located on Ring E, Me‐28, and Me‐30. This observation can be explained by the lack of stabilizing interactions 1,3‐di*axial* as present in methyl groups in Rings A and B.

## Conflicts of Interest

Armando Ariza‐Castolo is a guest editor of the special collection of Magnetic Resonance in Chemistry on NMR in Mexico and Central America. In accordance with the Journal's policy and COPE guidelines, the manuscript was handled by an independent editor to ensure a fair and unbiased review process.

## Supporting information


**Table S1:** Dihedral angles (*ϕ*) for taraxerone obtained from XRD, conformers **M** and **m** (B3LYP/6‐31G(d,p)), weighted conformers and using *J* values and HLA equation.
**Figure S1:** Correlation between *δ* ^1^H experimental and calculated using GIAO B3LYP/6‐31G(d,p).
**Figure S2:** Correlation between *δ* ^13^C experimental and calculated using GIAO B3LYP/6‐31G(d,p).
**Figure S3:** Correlation between ^1^J_C,H_ experimental and calculated using GIAO B3LYP/6‐31G(d,p).
**Figure S4:** NMR @ 500.16 MHz/CDCl_3_^1^H spectrum of taraxerone.
**Figure S5:** NMR @ 125.77 MHz/CDCl_3_^13^C spectrum of taraxerone.
**Figure S6:** NMR @ 125.77 MHz/CDCl_3_^13^C INEPT no‐decoupled spectrum of taraxerone.
**Figure S7:** NMR @ 500.16 MHz/CDCl_3_ HMBC spectrum of taraxerone.
**Figure S8:** NMR @ 500.16 MHz/CDCl_3_ ROESY experiment of taraxerone.
**Figure S9:** Left: NMR @ 500.16 MHz/CDCl_3_ *J*‐resolved experiment of taraxerone. Right: selected projections obtained from this experiment.
**Figure S10:** SS1 simulation of ^1^H NMR spectrum of taraxerone.
**Figure S11:** SS2 simulation of ^1^H NMR spectrum of taraxerone.
**Figure S12:** SS3 simulation of ^1^H NMR spectrum of taraxerone.
**Figure S13:** SS5 simulation of ^1^H NMR spectrum of taraxerone.
**Figure S14:** SS6 simulation of ^1^H NMR spectrum of taraxerone.
**Figure S15:** MS spectrum of taraxerone.
**Figure S16:** IR spectrum of taraxerone.
**Table S2:** Crystal data and structure refinement for taraxerone.
**Table S3:** Atomic coordinates (× 10^4^) and equivalent isotropic displacement parameters (pm^2^ × 10^−1^) for taraxerone. *U*(eq) is defined as one‐third of the trace of the orthogonalized *U* ^*ij*^ tensor.
**Table S4:** Bond lengths (pm) and angles (°) for taraxerone.
**Table S5:** Anisotropic displacement parameters (pm^2^ × 10^−1^). The anisotropic displacement factor exponent takes the form: −2^2^[*h* ^2^ *a* * ^2^ *U* ^11^ + … + 2*hka* * *b* * *U* ^12^].

## Data Availability

The data that support the findings of this study are available in the Supporting Information of this article.
