# Supplementary material for: Conformational Analysis and NMR Data Assignment of Taraxerone Isolated From Cnidoscolus aconitifolius (Chaya)
Source: Magn Reson Chem. 2025 Oct 22;64(1):146–59. doi: 10.1002/mrc.70055 (PMC12670066; doi:10.1002/mrc.70055)
Supplement: Supplementary file 1 — Table S1: Dihedral angles (ϕ) for taraxerone obtained from XRD, conformers M and m (B3LYP/6‐31G(d,p)), weighted conformers and using J values and HLA equation. Figure S1: Correlation between δ 1H experimental and calculated using GIAO B3LYP/6‐31G(d,p). Figure S2: Correlation between δ 13C experimental and calculated using GIAO B3LYP/6‐31G(d,p). Figure S3: Correlation between 1JC,H experimental and calculated using GIAO B3LYP/6‐31G(d,p). Figure S4: NMR @ 500.16 MHz/CDCl3 1H spectrum of taraxerone. Figure S5: NMR @ 125.77 MHz/CDCl3 13C spectrum of taraxerone. Figure S6: NMR @ 125.77 MHz/CDCl3 13C INEPT no‐decoupled spectrum of taraxerone. Figure S7: NMR @ 500.16 MHz/CDCl3 HMBC spectrum of taraxerone. Figure S8: NMR @ 500.16 MHz/CDCl3 ROESY experiment of taraxerone. Figure S9: Left: NMR @ 500.16 MHz/CDCl3 J‐resolved experiment of taraxerone. Right: selected projections obtained from this experiment. Figure S10: SS1 simulation of 1H NMR spectrum of taraxerone. Figure S11: SS2 simulation of 1H NMR spectrum of taraxerone. Figure S12: SS3 simulation of 1H NMR spectrum of taraxerone. Figure S13: SS5 simulation of 1H NMR spectrum of taraxerone. Figure S14: SS6 simulation of 1H NMR spectrum of taraxerone. Figure S15: MS spectrum of taraxerone. Figure S16: IR spectrum of taraxerone. Table S2: Crystal data and structure refinement for taraxerone. Table S3: Atomic coordinates (× 104) and equivalent isotropic displacement parameters (pm2 × 10−1) for taraxerone. U(eq) is defined as one‐third of the trace of the orthogonalized U ij tensor. Table S4: Bond lengths (pm) and angles (°) for taraxerone. Table S5: Anisotropic displacement parameters (pm2 × 10−1). The anisotropic displacement factor exponent takes the form: −22[h 2 a * 2 U 11 + … + 2hka * b * U 12]. [file MRC-64-146-s001.docx]

Electronic Supplementary Information File

**Conformational analysis and NMR data assignment of Taraxerone isolated from *Cnidoscolus aconitifolius* (Chaya)**

Mónica Díaz-Fernández,^a,b#^ Karla Kahun,^a#^ Viviana Roche-Llerena,^a^ Leonardo Hernández,^a^ Geonel Rodríguez-Gattorno,^a^ Armando Ariza-Castolo,^b^* María A. Fernández-Herrera.^a^*

# authors contributed equally to this work

^a^Departamento de Física Aplicada, Centro de Investigación y de Estudios Avanzados del Instituto Politécnico Nacional, Unidad Mérida. Km 6 Antigua carretera a Progreso. Apdo. Postal 73, Cordemex, 97310, Mérida, Yuc., México.

^b^Departamento de Química, Centro de Investigación y de Estudios Avanzados del Instituto Politécnico Nacional, Unidad Zacatenco. Avenida Instituto Politécnico Nacional 2508 Colonia San Pedro Zacatenco, 07360, Ciudad de México, México

Content

| Table S1. Dihedral angles (φ) for taraxerone obtained from XRD, conformers **M** and **m** (B3LYP/6-31G(d,p)), weighted conformers, and using J values and HLA equation | S2 |
| --- | --- |
| Figure S1. Correlation between δ ^1^H experimental and calculated using GIAO B3LYP/6-31G(d,p). | S3 |
| Figure S2. Correlation between δ ^13^C experimental and calculated using GIAO B3LYP/6-31G(d,p). | S3 |
| Figure S3. Correlation between ^1^J_C,H_ experimental and calculated using GIAO B3LYP/6-31G(d,p). | S4 |
| Figure S4. NMR @500.16 MHz/CDCl_3_ ^1^H spectrum of taraxerone. | S5 |
| Figure S5. NMR @125.77 MHz/CDCl_3_ ^13^C spectrum of taraxerone. | S6 |
| Figure S6. NMR @125.77 MHz/CDCl_3_ ^13^C INEPT-no decoupled spectrum of taraxerone. | S7 |
| Figure S7. NMR @500.16 MHz/CDCl_3_ HMBC spectrum of taraxerone. | S8 |
| Figure S8. NMR @500.16 MHz/CDCl_3_ ROESY experiment of taraxerone., | S9 |
| Figure S9. Left: NMR @500.16 MHz/CDCl_3_ *J* resolved experiment of taraxerone. Right: selected projections obtained from this experiment. | S10 |
| Figure S10.SS1 simulation of ^1^H NMR spectrum of taraxerone. | S11 |
| Figure S11.SS2 simulation of ^1^H NMR spectrum of taraxerone. | S12 |
| Figure S12.SS3 simulation of ^1^H NMR spectrum of taraxerone. | S13 |
| Figure S13.SS5 simulation of ^1^H NMR spectrum of taraxerone. | S14 |
| Figure S14.SS6 simulation of ^1^H NMR spectrum of taraxerone. | S15 |
| Figure S15. MS spectrum of taraxerone. | S16 |
| Figure S16. IR spectrum of taraxerone. | S16 |
| Optimized geometry of Conformer **m**. | S17 |
| Optimized geometry of Conformer **M**. | S19 |
| Table S2. Crystal data and structure refinement for taraxerone. | S21 |
| Table S3. Atomic coordinates (x 104) and equivalent isotropic displacementparameter. | S22 |
| Table S4. Bond lengths [pm] and angles [°] for taraxerone. | S23 |
| Table S5. Anisotropic displacement parameters. | S30 |

Table S1. Dihedral angles (φ) for taraxerone obtained from XRD, conformers **M** and **m** (B3LYP/6-31G(d,p)), weighted conformers and using J values and HLA equation.

| φ (°) | **XRD** | **M** | **m** | **weighted** | **HLA** |
| --- | --- | --- | --- | --- | --- |
| 1*_ax_*-2*_ax_* | 171.4 | 171.8 | 91.4 | 161.1 | 155.4 |
| 1*_ax_*-2*_eq_* | 53.5 | -53.5 | 27.1 | -42.8 | 47.8 |
| 1*_eq_*-2*_ax_* | 55.5 | -56.3 | 123.6 | -32.6 | 42.4 |
| 1*_eq_*-2*_eq_* | 62.3 | 62.0 | 142.1 | 72.5 | 64.0 |
| 5*_ax_*-6*_ax_* | 174.1 | 174.4 | 179.1 | 175.0 | 159.9 |
| 5*_ax_*-6*_eq_* | 68.9 | 68.8 | 64.8 | 68.2 | 58.5 |
| 6*_ax_*-7*_ax_* | 172.2 | 171.8 | 172.9 | 171.9 | 170.9 |
| 6*_ax_*-7*_eq_* | 56.4 | 56.3 | 57.1 | 56.4 | 61.0 |
| 6*_eq_*-7*_ax_* | 56.6 | 56.4 | 57.6 | 56.6 | 53.7 |
| 6*_eq_*-7*_eq_* | 59.2 | 59.0 | 58.2 | 58.9 | 61.9 |
| 9*_ax_*-11*_ax_* | 147.8 | 148.7 | 150.7 | 148.9 | 155.5 |
| 9*_ax_*-11*_eq_* | 33.3 | 33.4 | 35.5 | 33.7 | 33.1 |
| 11*_ax_*-12*_ax_* | 149.8 | 149.7 | 148.3 | 149.5 | 144.5 |
| 11*_ax_*-12*_eq_* | 34.2 | 34.5 | 33.2 | 34.3 | 27.4 |
| 11*_eq_*-12*_ax_* | 35.4 | 35.6 | 34.2 | 35.4 | 28.7 |
| 11*_eq_*-12*_eq_* | 80.2 | 79.6 | 80.9 | 79.8 | 90.0 |
| 18*_ax_*-19*_ax_* | 179.8 | 179.8 | 179.7 | 179.8 | 180.0 |
| 18*_ax_*-19*_eq_* | 64.1 | 64.0 | 64.0 | 64.0 | 58.7 |
| 21*_ax_*-22*_eq_* | 60.2 | 59.9 | 59.9 | 59.9 | 62.3 |
| 21*_ax_*-22*_ax_* | 176.5 | 175.8 | 175.8 | 175.8 | 168.3 |
| 21*_eq_*-22*_eq_* | 55.3 | 55.4 | 55.4 | 55.4 | 60.5 |
| 21*_eq_*-22*_ax_* | 60.9 | 60.5 | 60.5 | 60.5 | 64.4 |

Figure S1. Correlation between δ ^1^H experimental and calculated using GIAO B3LYP/6-31G(d,p).

Figure S2. Correlation between δ ^13^C experimental and calculated using GIAO B3LYP/6-31G(d,p).

Figure S3. Correlation between ^1^*J*_C,H_ experimental and calculated using GIAO B3LYP/6-31G(d,p).

Figure S4. NMR @500.16 MHz/CDCl_3_ ^1^H spectrum of taraxerone.

Figure S5. NMR @125.77 MHz/CDCl_3_ ^13^C spectrum of taraxerone.

** Figure S6. NMR @125.77 MHz/CDCl_3_ ^13^C INEPT no-decoupled spectrum of taraxerone.


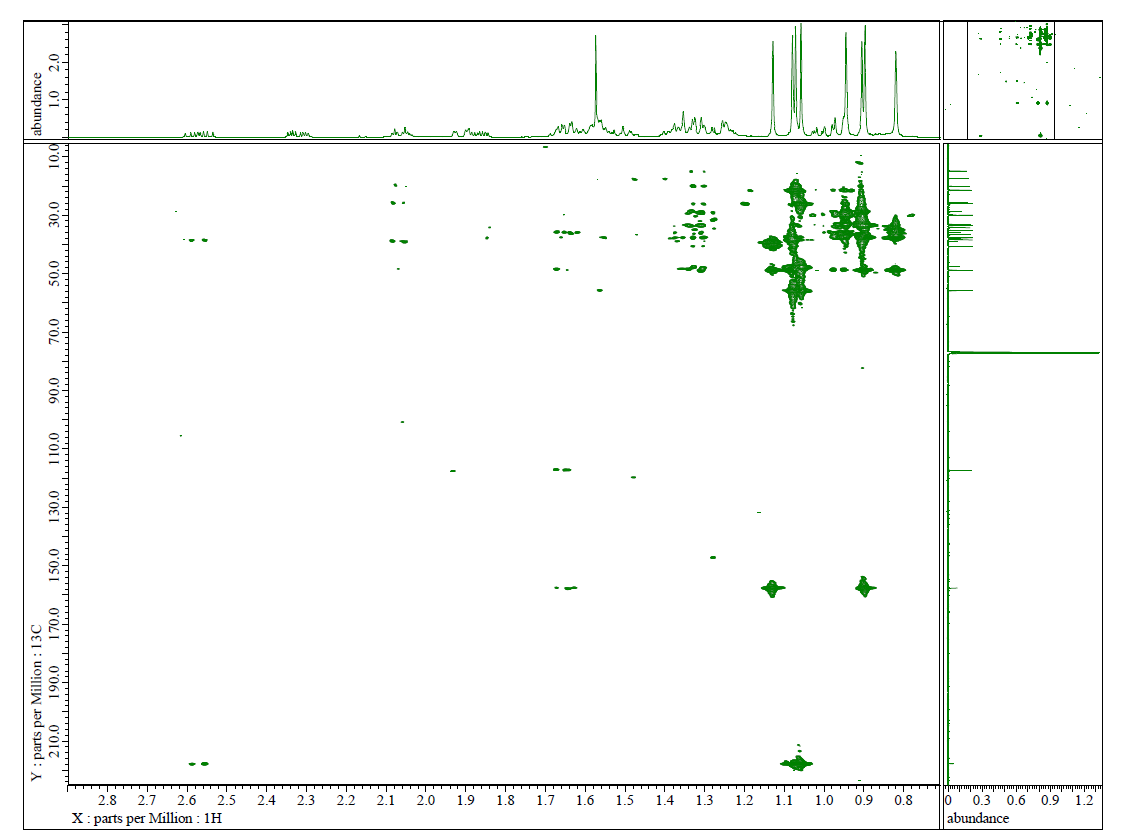


Figure S7. NMR @500.16 MHz/CDCl_3_ HMBC spectrum of taraxerone.

Figure S8. NMR @500.16 MHz/CDCl_3_ ROESY experiment of taraxerone.


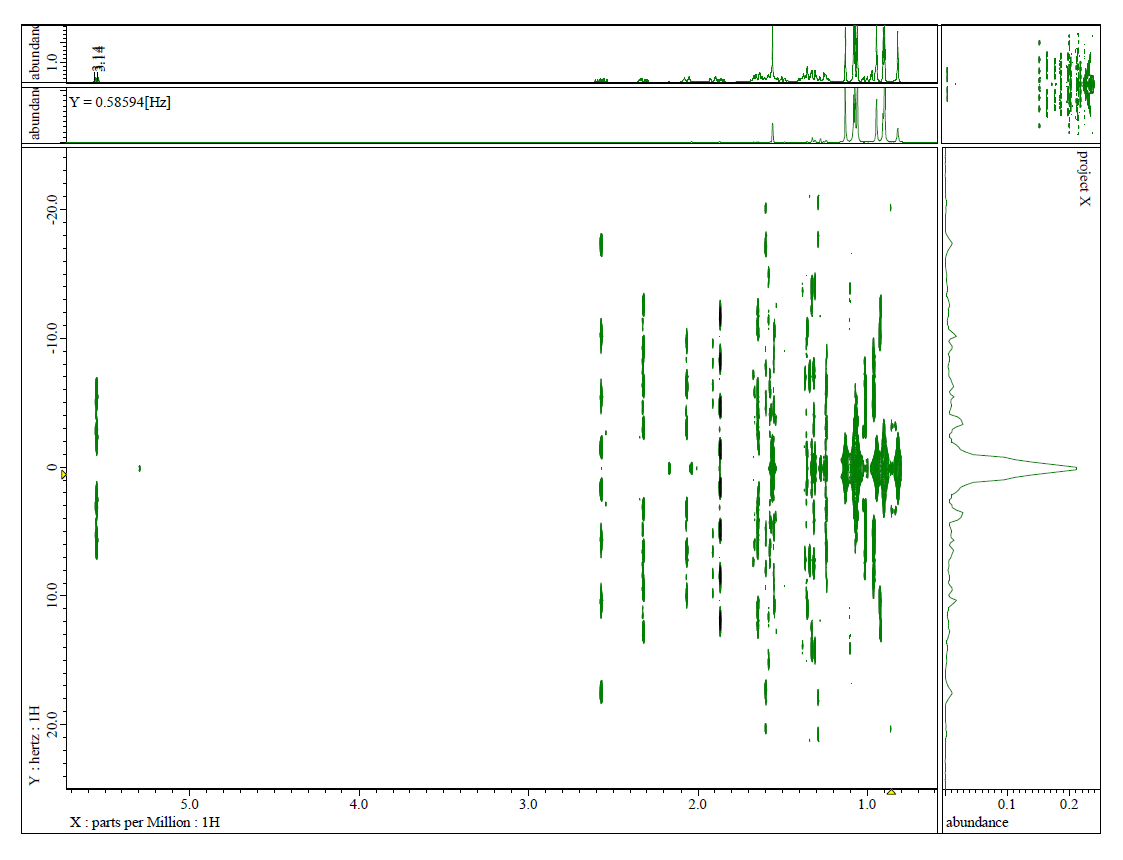

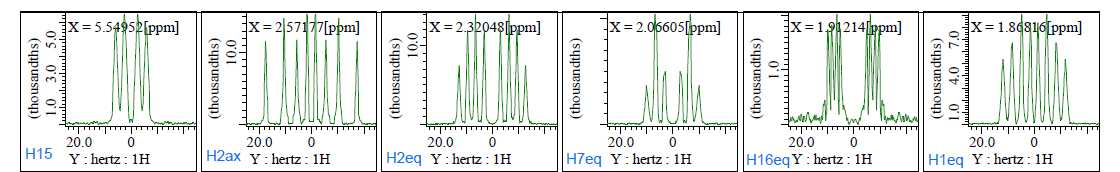

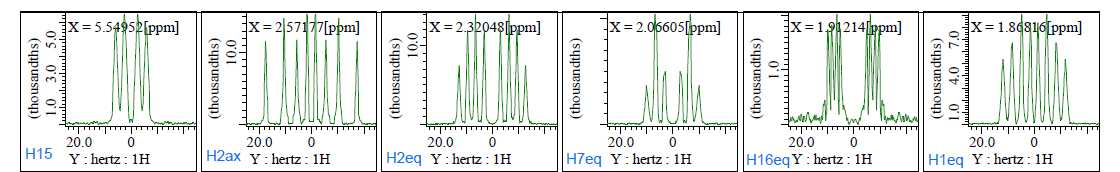


Figure S9. Left: NMR @500.16 MHz/CDCl_3_ *J* resolved experiment of taraxerone. Right: selected projections obtained from this experiment.


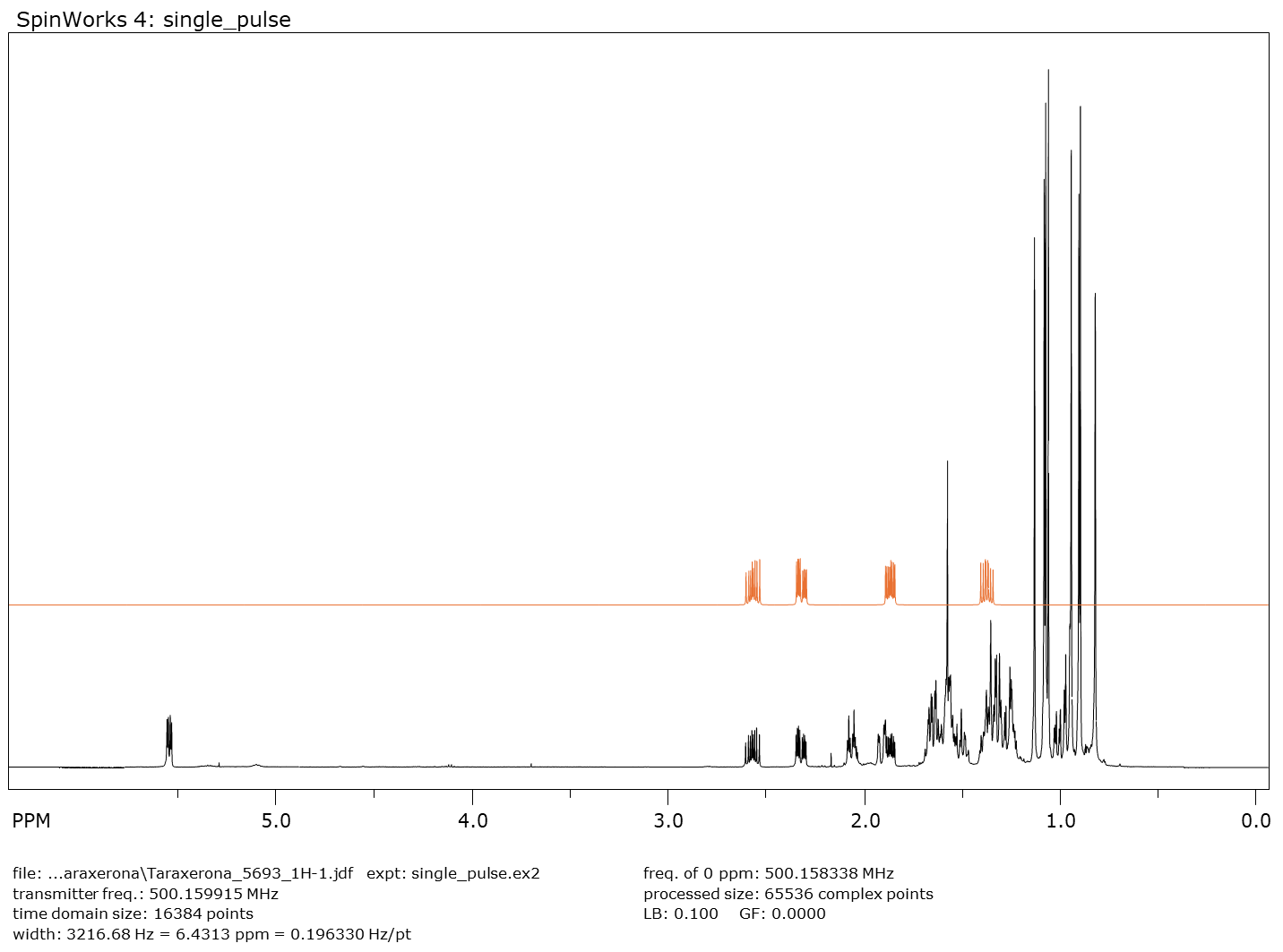

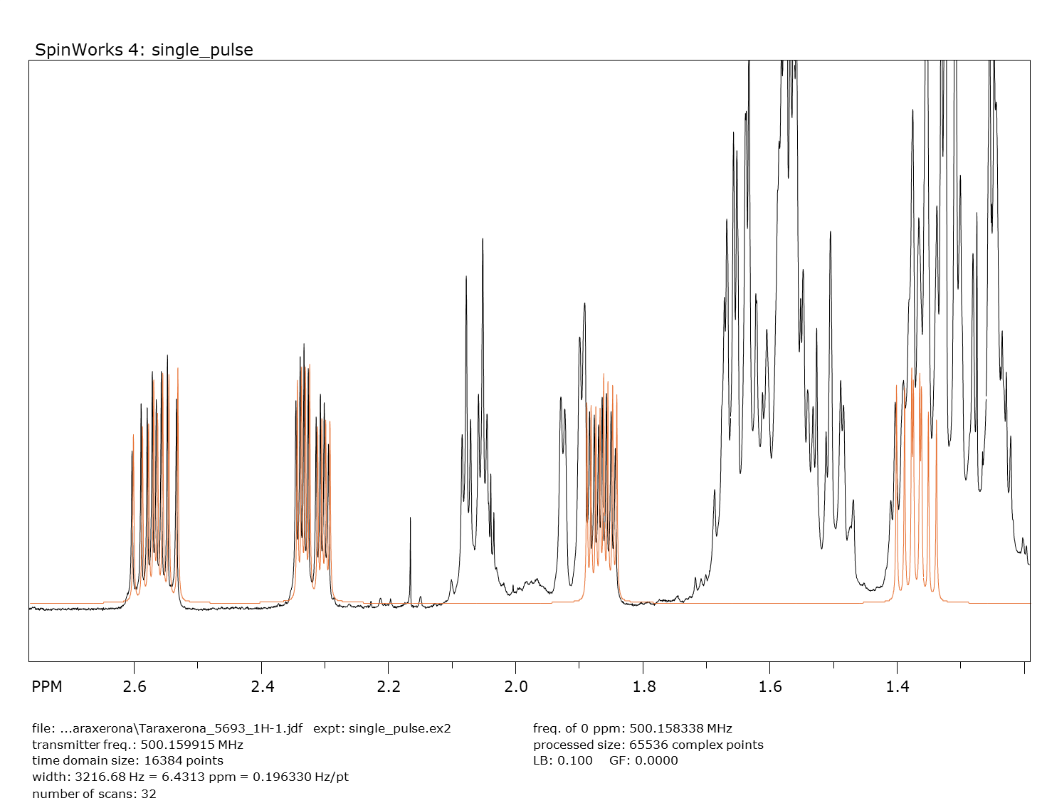


Figure S10.SS1 simulation of ^1^H NMR spectrum of taraxerone.


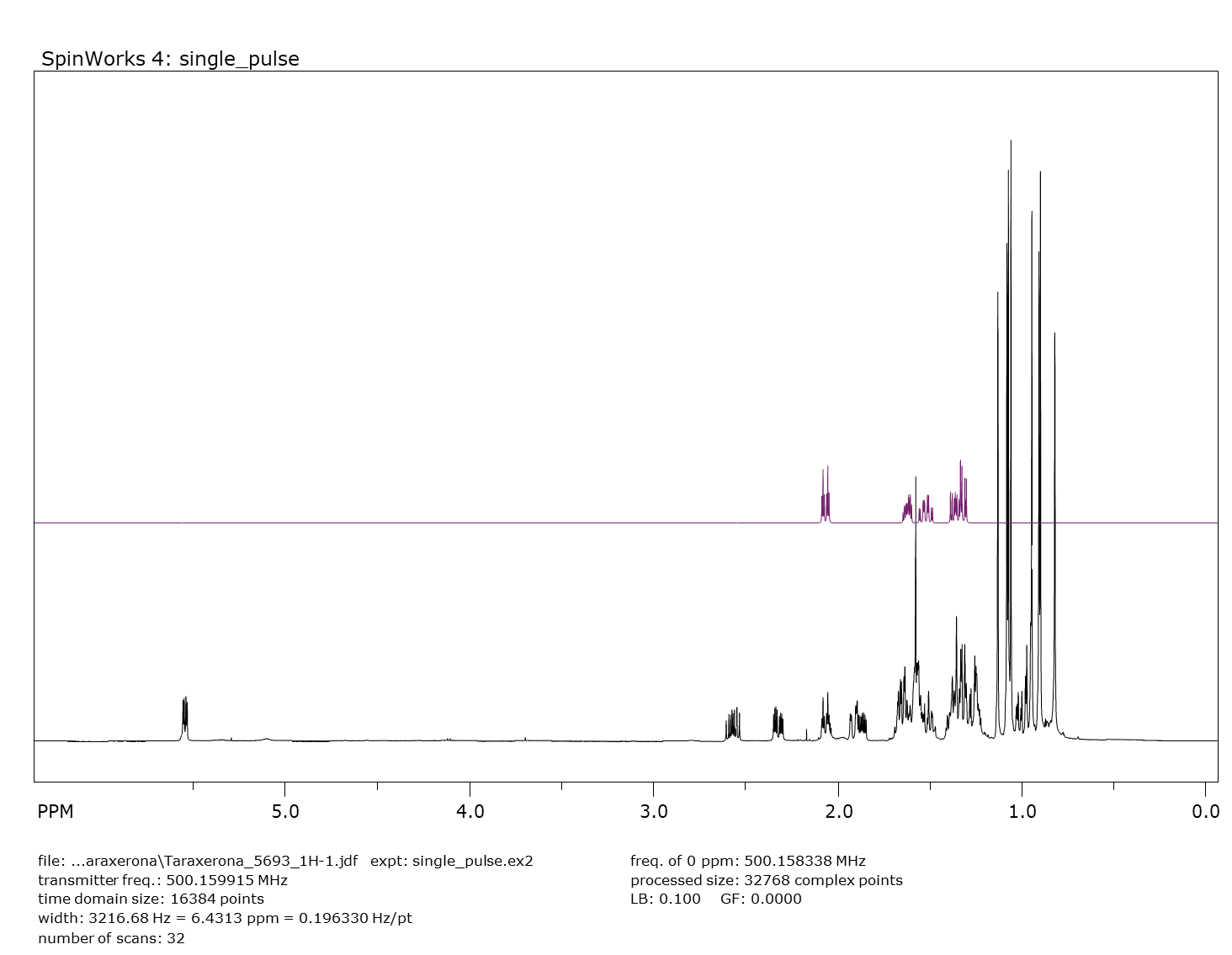

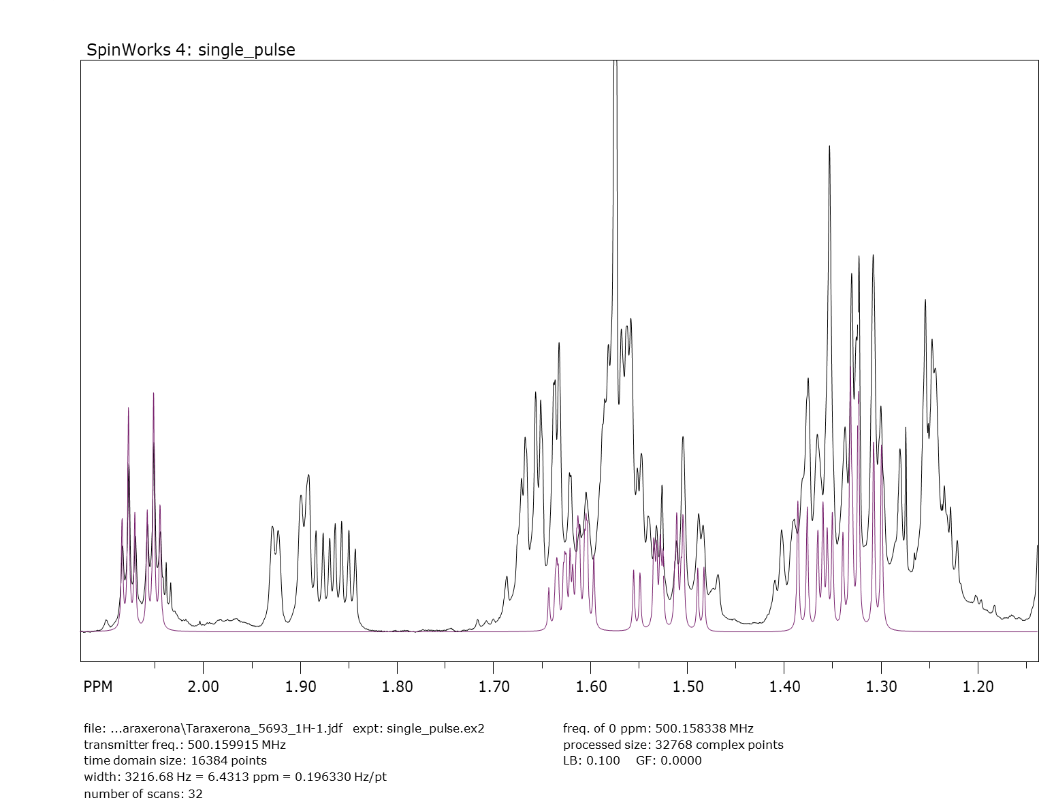


Figure S11.SS2 simulation of ^1^H NMR spectrum of taraxerone.


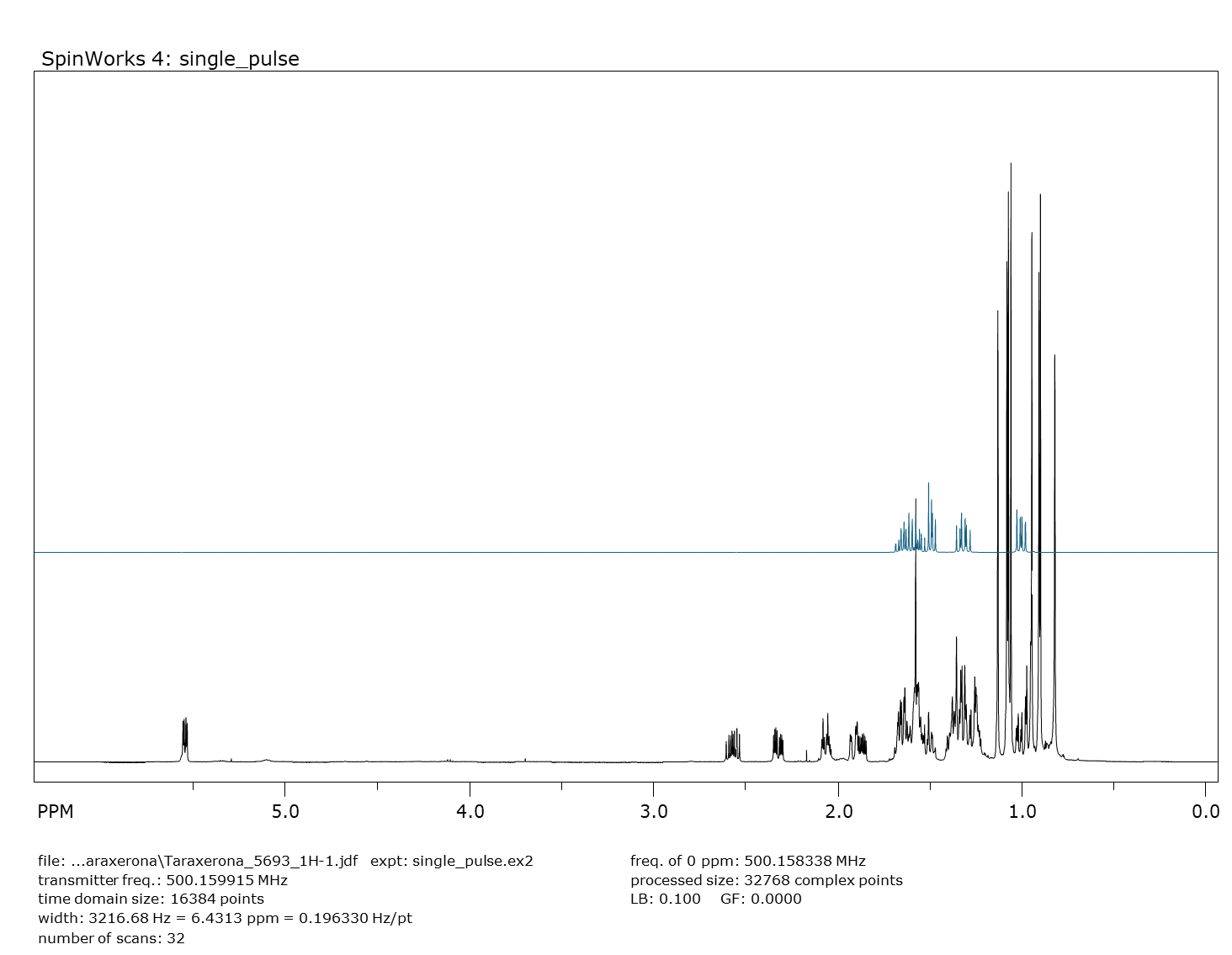

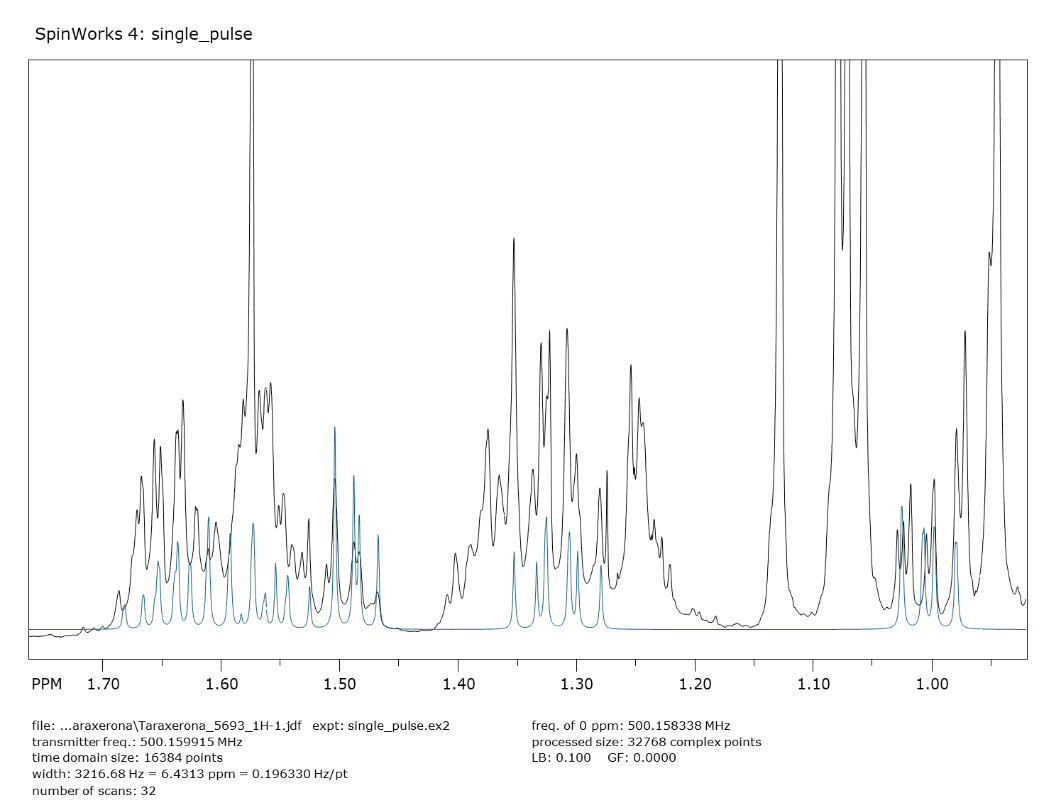


Figure S12.SS3 simulation of ^1^H NMR spectrum of taraxerone.


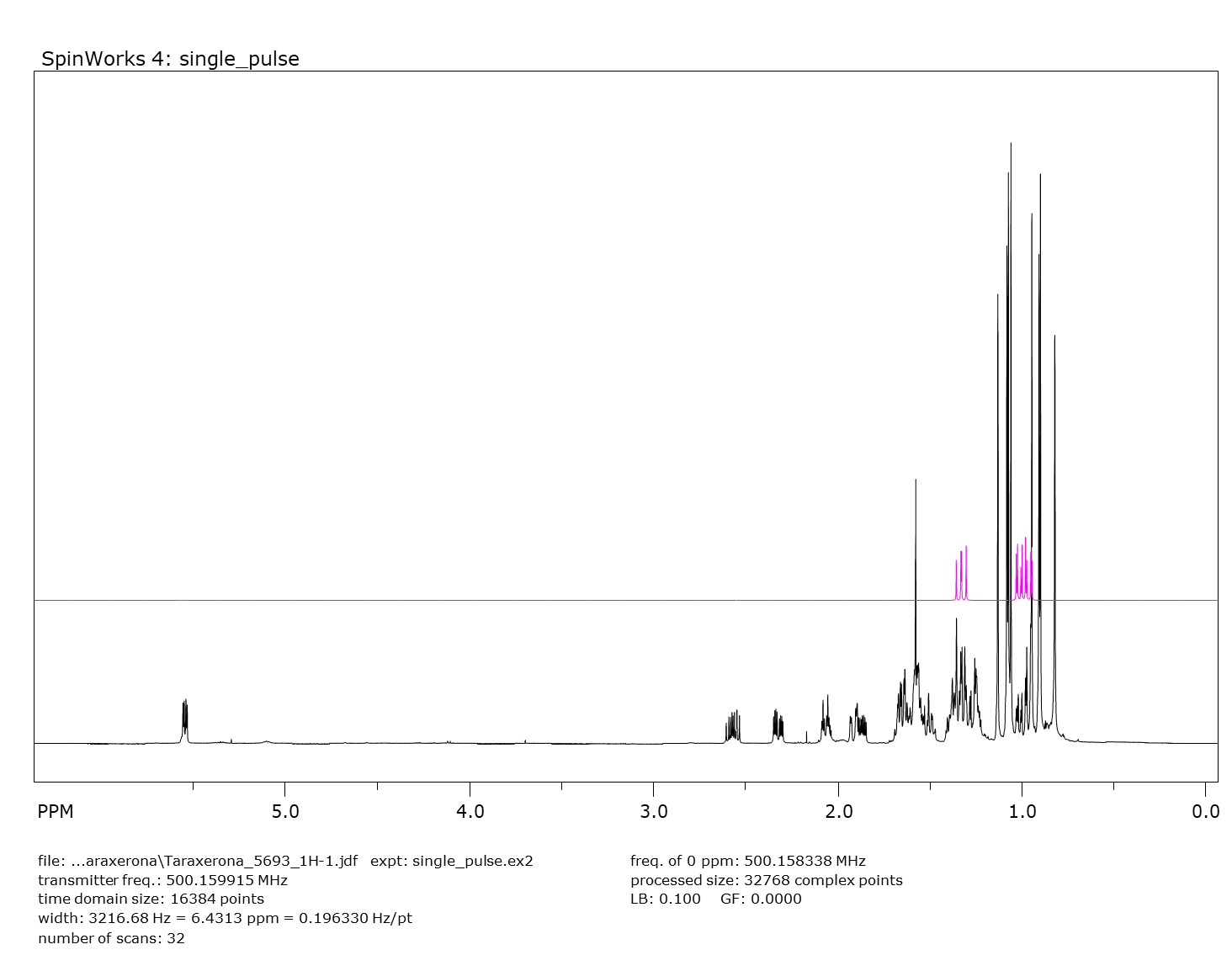

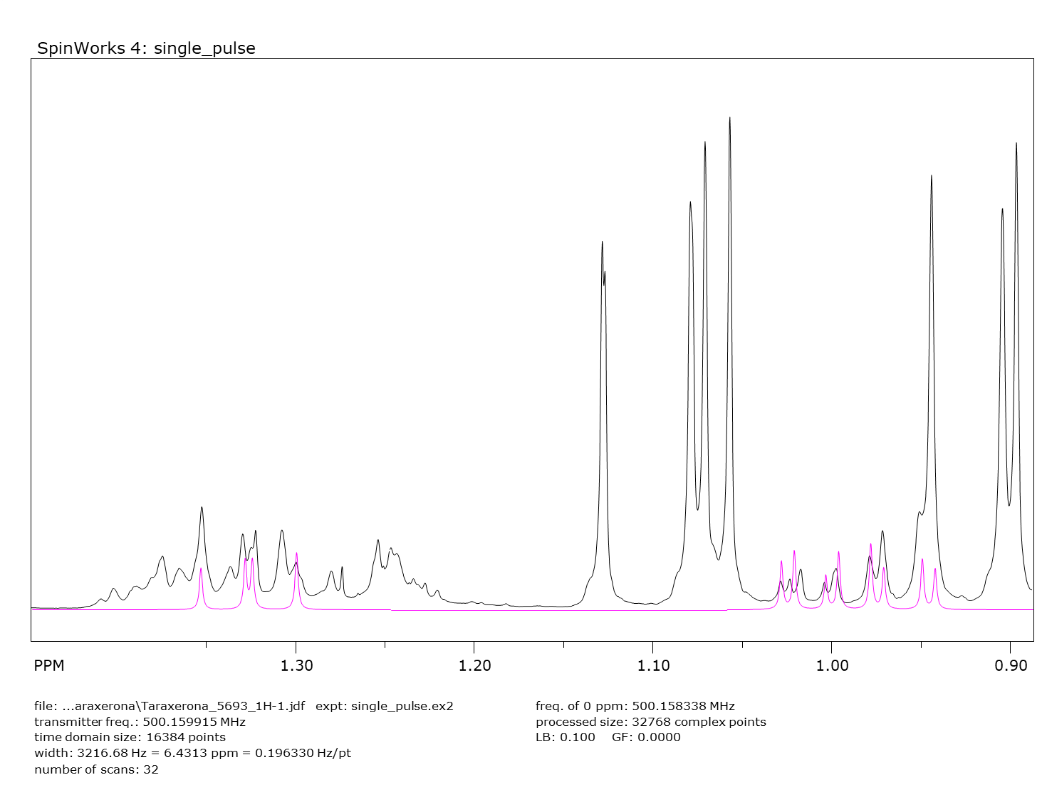


Figure S13.SS5 simulation of ^1^H NMR spectrum of taraxerone.


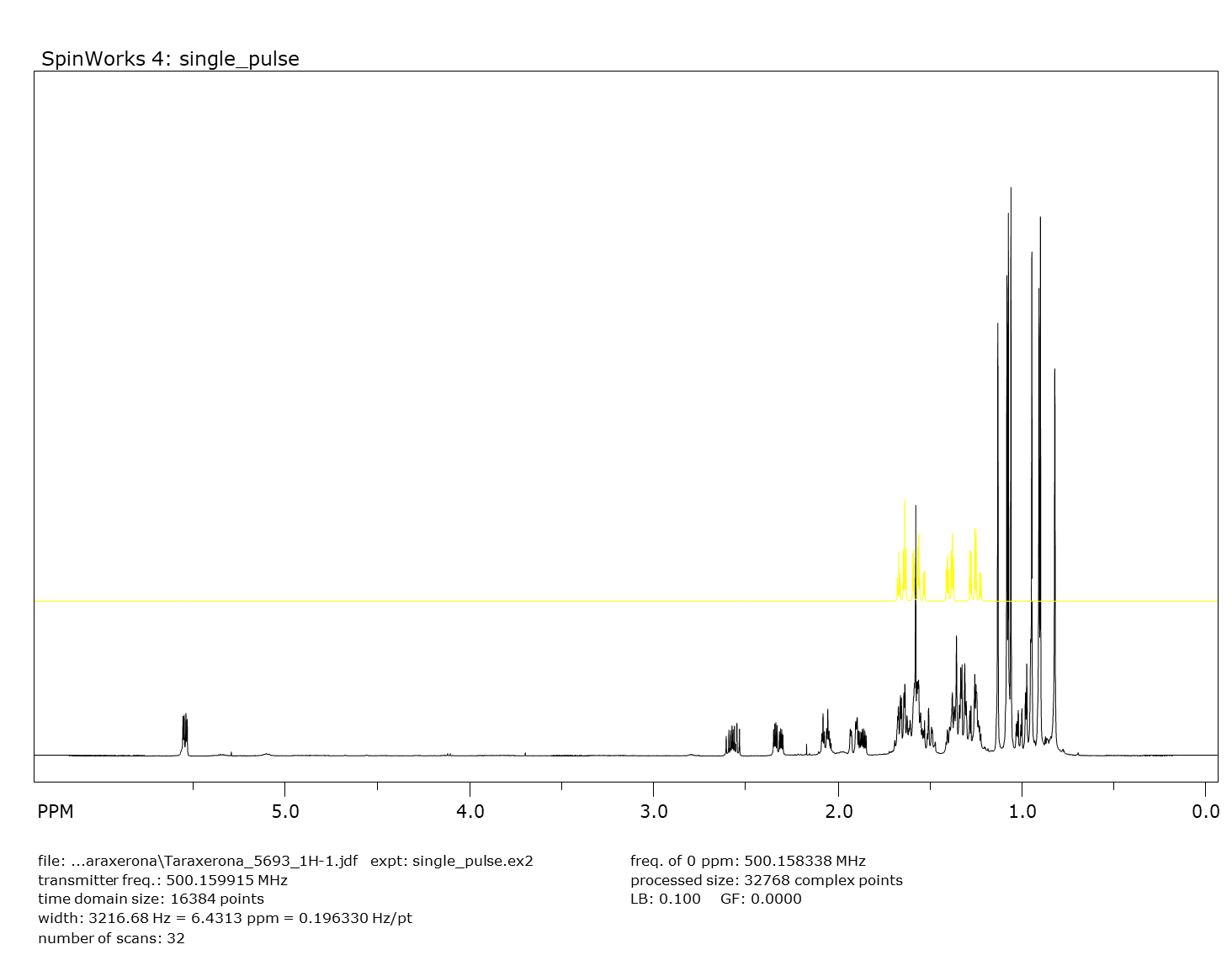

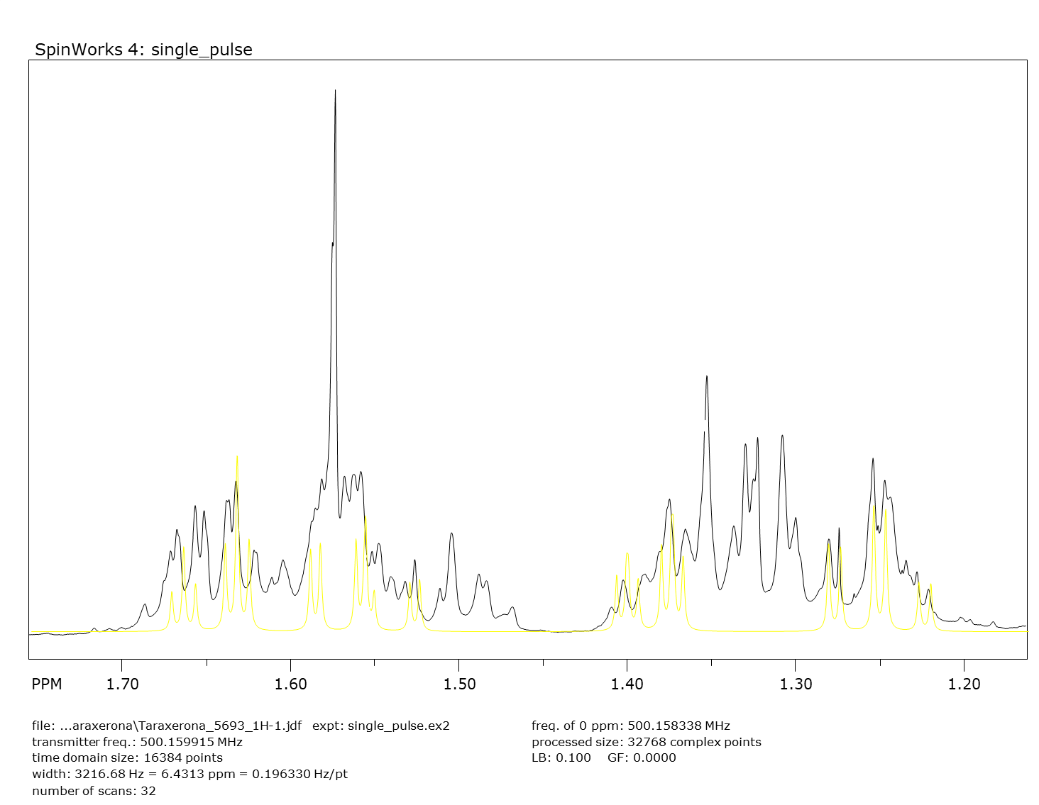


Figure S14.SS6 simulation of ^1^H NMR spectrum of taraxerone.


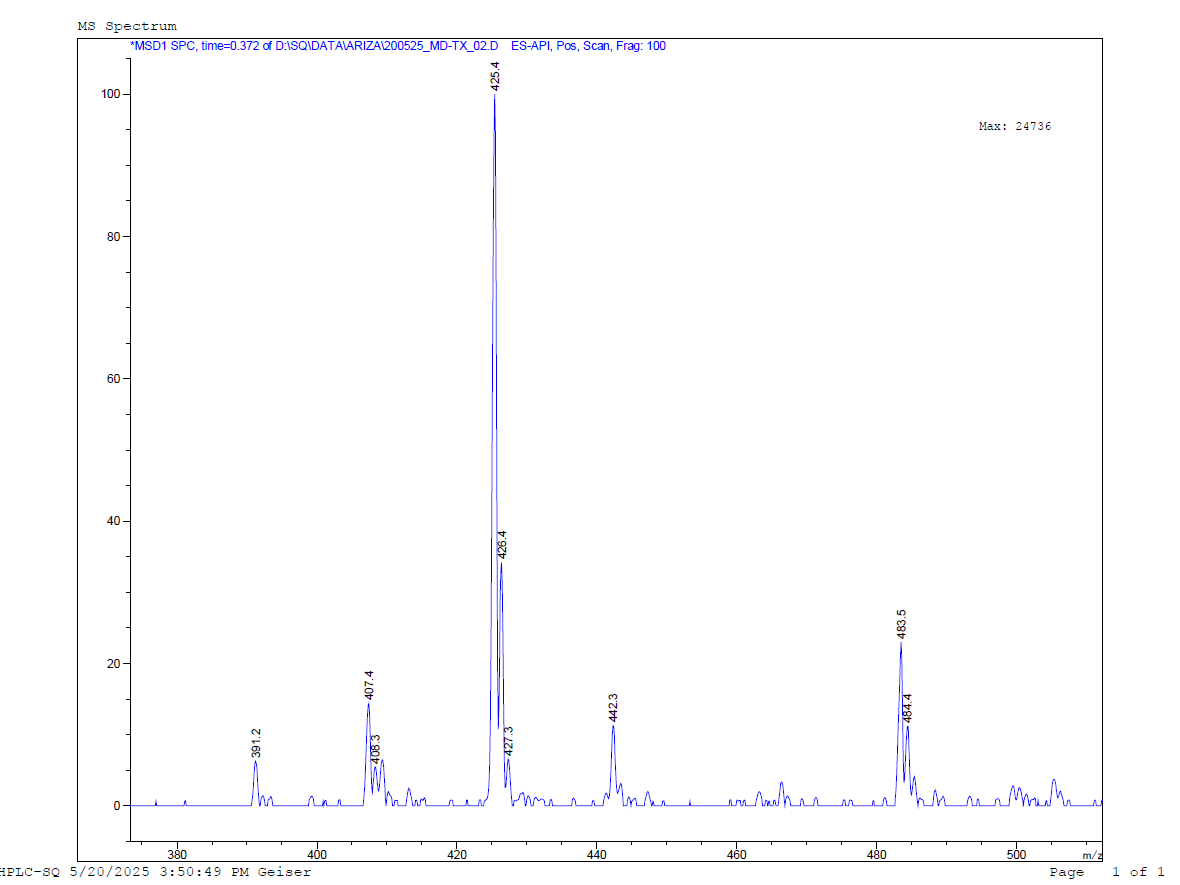


Figure S15. MS spectrum of taraxerone.

Figure S16.IR spectrum of taraxerone.

Optimized geometry of Conformer **M**.

| C | -0.83624271 | 0.05719996 | 3.03731995 |
| --- | --- | --- | --- |
| C | -2.69770045 | 0.37846259 | 4.79760248 |
| C | -1.2614343 | -1.80101288 | 4.9280945 |
| C | -2.58833984 | -1.07108438 | 5.23527318 |
| C | -0.81150182 | -1.44971801 | 3.46292662 |
| C | -2.25475795 | 0.60300358 | 3.34341637 |
| H | -2.08479083 | 0.98093609 | 5.4779292 |
| H | -1.58916413 | -1.91593316 | 2.83948864 |
| H | -2.97495745 | 0.1076614 | 2.68087374 |
| H | -3.73347536 | 0.68797556 | 4.948902 |
| H | -2.31137147 | 1.67168732 | 3.11940863 |
| O | -3.50152778 | -1.61578224 | 5.81639561 |
| C | -0.24716952 | -1.39870188 | 6.02891304 |
| H | -0.08736444 | -0.3238226 | 6.10095357 |
| H | 0.72136263 | -1.86928674 | 5.8431227 |
| H | -0.60508875 | -1.75020655 | 7.00019144 |
| C | -1.50997307 | -3.31714849 | 5.05033008 |
| H | -2.01336918 | -3.53466069 | 5.99212059 |
| H | -0.57323044 | -3.87682505 | 5.0233985 |
| H | -2.15314292 | -3.68153526 | 4.24468852 |
| C | -0.64368382 | 0.08173013 | 1.47046049 |
| H | -1.46819762 | -0.54639911 | 1.11231627 |
| C | 0.50858384 | -2.10875006 | 3.03570836 |
| H | 1.35868289 | -1.63476462 | 3.53537453 |
| H | 0.52713134 | -3.15630474 | 3.34489003 |
| C | 0.62644967 | -0.63860376 | 0.90234558 |
| C | 0.68413032 | -2.06291424 | 1.51305916 |
| H | -0.10426846 | -2.67144011 | 1.05709212 |
| H | 1.63427131 | -2.53826365 | 1.25108617 |
| C | -0.87227329 | 1.48339659 | 0.85037224 |
| H | -0.50049463 | 2.26909658 | 1.51363802 |
| H | -1.94944161 | 1.65243088 | 0.7737631 |
| C | 0.2032409 | 0.93655626 | 3.77681987 |
| H | 0.4680488 | 1.81551415 | 3.18711404 |
| H | 1.12858637 | 0.40858112 | 4.00123949 |
| H | -0.18524412 | 1.30928017 | 4.72540183 |
| C | 1.97039002 | 0.0737484 | 1.22338974 |
| H | 1.96492204 | 1.1339505 | 0.97033803 |
| H | 2.76976273 | -0.39861786 | 0.6477947 |
| H | 2.23355528 | -0.00724572 | 2.2776289 |
| C | -0.2284976 | 1.69136646 | -0.53260103 |
| H | 0.79419757 | 2.05713637 | -0.41141772 |
| H | -0.76685237 | 2.48644921 | -1.05743116 |
| C | 0.48978373 | -0.72094025 | -0.63913187 |
| C | 0.93981093 | -1.76571241 | -1.34168416 |
| H | 1.38483142 | -2.62064157 | -0.84638155 |
| C | -0.18626032 | 0.42494706 | -1.41359289 |
| C | 0.61258903 | 0.78253558 | -2.74129897 |
| H | 1.36761613 | 1.50539786 | -2.41728943 |
| C | 1.41086836 | -0.4057408 | -3.40486173 |
| C | 0.92967797 | -1.76645408 | -2.84577265 |
| H | 1.57629522 | -2.5629002 | -3.22887196 |
| H | -0.07310874 | -1.98646682 | -3.23324441 |
| C | -1.64577742 | -0.01368846 | -1.72671896 |
| H | -2.22496712 | 0.80546645 | -2.15923108 |
| H | -2.15998561 | -0.3323291 | -0.81965596 |
| H | -1.67805137 | -0.85274182 | -2.42292567 |
| C | -0.27739427 | 1.50651957 | -3.77251395 |
| H | -0.80636987 | 2.33653831 | -3.29291214 |
| H | -1.05381228 | 0.82085615 | -4.12375233 |
| C | 1.23473726 | -0.39480344 | -4.94260558 |
| H | 0.23012877 | -0.75013011 | -5.1997989 |
| H | 1.93083959 | -1.11749453 | -5.38357193 |
| C | 0.46722499 | 2.0628091 | -5.01363403 |
| C | 1.46124626 | 0.98911672 | -5.55989339 |
| H | 2.49014626 | 1.30475905 | -5.36283965 |
| H | 1.37500085 | 0.92555371 | -6.64935722 |
| C | 2.91308035 | -0.25921254 | -3.07417378 |
| H | 3.07999357 | -0.27986956 | -1.99575212 |
| H | 3.48977285 | -1.07567714 | -3.52160275 |
| H | 3.32030384 | 0.68228885 | -3.45066775 |
| C | -0.58297896 | 2.39439942 | -6.09045836 |
| H | -1.3321652 | 3.09716343 | -5.71123483 |
| H | -1.10816955 | 1.49212911 | -6.41964157 |
| H | -0.11313187 | 2.84744759 | -6.96915397 |
| C | 1.22819685 | 3.35269712 | -4.65648981 |
| H | 0.54080088 | 4.13716184 | -4.32342942 |
| H | 1.77241578 | 3.73394197 | -5.52635903 |
| H | 1.95805189 | 3.18593242 | -3.85922835 |

Optimized geometry of Conformer **m**.

| C | -0.84193801 | -0.08826092 | 3.03278389 |
| --- | --- | --- | --- |
| C | -2.96627211 | -0.33435651 | 4.5477645 |
| C | -0.93804559 | -1.86996572 | 4.98100722 |
| C | -1.96175413 | -0.86812715 | 5.54785903 |
| C | -0.69556578 | -1.58615698 | 3.45274627 |
| C | -2.31117232 | 0.35826309 | 3.32496747 |
| H | -3.65259419 | 0.33087512 | 5.07250219 |
| H | -1.50309925 | -2.10694988 | 2.91811853 |
| H | -2.93664565 | 0.15912466 | 2.45062452 |
| H | -3.54927365 | -1.19472567 | 4.19912541 |
| H | -2.33220211 | 1.44034793 | 3.47572169 |
| O | -1.99223374 | -0.56489579 | 6.72081517 |
| C | 0.32858453 | -1.85738946 | 5.85793705 |
| H | 0.89340289 | -0.92965635 | 5.76488847 |
| H | 0.99022304 | -2.68734208 | 5.60179647 |
| H | 0.03735933 | -1.95752085 | 6.90383178 |
| C | -1.59789573 | -3.26793931 | 5.13162548 |
| H | -1.76330572 | -3.50080703 | 6.18609934 |
| H | -0.94626986 | -4.03916247 | 4.71220955 |
| H | -2.55879527 | -3.32632414 | 4.61316686 |
| C | -0.63421204 | 0.00817096 | 1.47222992 |
| H | -1.44425587 | -0.62223462 | 1.08511159 |
| C | 0.61231028 | -2.19901275 | 2.9351356 |
| H | 1.47243217 | -1.72043784 | 3.41039307 |
| H | 0.66434669 | -3.25446704 | 3.22199315 |
| C | 0.64511637 | -0.65791462 | 0.86462102 |
| C | 0.72943387 | -2.10528682 | 1.41250001 |
| H | -0.07126651 | -2.69890902 | 0.95768959 |
| H | 1.67249028 | -2.56536815 | 1.10274467 |
| C | -0.8890911 | 1.43302854 | 0.91940261 |
| H | -0.50036623 | 2.18907619 | 1.60550112 |
| H | -1.96864602 | 1.60119117 | 0.88207514 |
| C | 0.09594317 | 0.82797594 | 3.85178352 |
| H | 0.10032082 | 1.84521867 | 3.4557721 |
| H | 1.12735657 | 0.4797328 | 3.87137647 |
| H | -0.23953482 | 0.90355423 | 4.88880052 |
| C | 1.9719077 | 0.07333065 | 1.20942506 |
| H | 1.93209168 | 1.1442741 | 1.00927936 |
| H | 2.78196913 | -0.3462267 | 0.60832936 |
| H | 2.24415328 | -0.04952531 | 2.25762152 |
| C | -0.2798909 | 1.70739636 | -0.46865086 |
| H | 0.73623219 | 2.09270937 | -0.35183764 |
| H | -0.84637561 | 2.50871542 | -0.95288923 |
| C | 0.49421471 | -0.67916745 | -0.67800306 |
| C | 0.96172409 | -1.68394665 | -1.42603796 |
| H | 1.43457611 | -2.54552766 | -0.969792 |
| C | -0.22116429 | 0.47772406 | -1.39882795 |
| C | 0.54969458 | 0.90554223 | -2.72277739 |
| H | 1.29061036 | 1.63491034 | -2.38123757 |
| C | 1.36928219 | -0.2351591 | -3.4418646 |
| C | 0.93269591 | -1.62814793 | -2.92851816 |
| H | 1.59567469 | -2.39159476 | -3.3491208 |
| H | -0.06857071 | -1.86130821 | -3.31258021 |
| C | -1.67411652 | 0.01718211 | -1.71002098 |
| H | -2.27620276 | 0.8388421 | -2.10458067 |
| H | -2.16899497 | -0.34562073 | -0.80886011 |
| H | -1.69736223 | -0.79602764 | -2.43675891 |
| C | -0.37202208 | 1.64527513 | -3.71426887 |
| H | -0.91416399 | 2.44335522 | -3.19671107 |
| H | -1.13639329 | 0.95444825 | -4.08113154 |
| C | 1.16956405 | -0.17234025 | -4.97541565 |
| H | 0.17007775 | -0.54337934 | -5.23040734 |
| H | 1.87656417 | -0.86060995 | -5.45262992 |
| C | 0.34057053 | 2.26563868 | -4.94401438 |
| C | 1.35214311 | 1.23842439 | -5.54411901 |
| H | 2.37593468 | 1.57230765 | -5.35066562 |
| H | 1.25067467 | 1.21282969 | -6.6338083 |
| C | 2.87187534 | -0.0601799 | -3.12668907 |
| H | 3.24793092 | 0.90572503 | -3.47274525 |
| H | 3.46353152 | -0.84304061 | -3.6131571 |
| H | 3.05555169 | -0.1171724 | -2.05226954 |
| C | -0.73385412 | 2.61001138 | -5.99270706 |
| H | -1.49383234 | 3.27988351 | -5.57714727 |
| H | -1.2422052 | 1.70754662 | -6.34698703 |
| H | -0.28873234 | 3.10599106 | -6.86102489 |
| C | 1.07432777 | 3.56044643 | -4.54984852 |
| H | 0.37273568 | 4.3139821 | -4.17747641 |
| H | 1.59516329 | 3.98803444 | -5.41246655 |
| H | 1.82052531 | 3.38310012 | -3.77032782 |

Table S2. Crystal data and structure refinement for taraxerone.

Identification code shelx

Empirical formula C_30_ H_48_ O

Formula weight 424.68

Temperature 293(2) K

Wavelength 71.073 pm

Crystal system Triclinic

Space group P 1

Unit cell dimensions a = 622.90(12) pm α= 90.80(3)°.

b = 735.44(15) pm β= 97.34(3)°.

c = 1513.7(3) pm γ = 113.63(3)°.

Volume 0.6285(3) nm3

Z 1

Density (calculated) 1.122 Mg/m3

Absorption coefficient 0.065 mm-1

F(000) 236

Crystal size 0.40 x 0.15 x 0.12 mm3

Theta range for data collection 2.720 to 25.288°.

Index ranges -6<=h<=6, -8<=k<=8, -16<=l<=16

Reflections collected 2287

Independent reflections 2287 [R(int) = 0.0124]

Completeness to theta = 25.242° 100.0 %

Refinement method Full-matrix least-squares on F2

Data / restraints / parameters 2162 / 3 / 288

Goodness-of-fit on F2 1.089

Final R indices [I>2sigma(I)] R1 = 0.0451, wR2 = 0.1183

R indices (all data) R1 = 0.0475, wR2 = 0.1209

Absolute structure parameter 5(3)

Extinction coefficient n/a

Largest diff. peak and hole 0.0244 and -0.0191 e.pmTable S3. Atomic coordinates ( x 10^4^) and equivalent isotropic displacement parameters (pm^2^x 10^–1^) for taraxerone. U(eq) is defined as one third of the trace of the orthogonalized U*ij* tensor.

_____________________________________________________________________

x y z U(eq)

_____________________________________________________________________

C(1) 8013(6) 6608(4) 5736(2) 48(1)

C(2) 8546(7) 6542(5) 6752(2) 58(1)

C(3) 10686(6) 8366(5) 7132(2) 51(1)

C(4) 10629(5) 10384(4) 6941(2) 44(1)

C(5) 9818(4) 10324(4) 5914(2) 37(1)

C(6) 9640(5) 12232(4) 5613(2) 45(1)

C(7) 9643(5) 12342(4) 4606(2) 43(1)

C(8) 7644(4) 10557(3) 4043(2) 36(1)

C(9) 7630(4) 8605(3) 4438(2) 33(1)

C(10) 7623(4) 8476(4) 5473(2) 37(1)

C(11) 5769(5) 6721(4) 3890(2) 44(1)

C(12) 5076(5) 7024(4) 2914(2) 39(1)

C(13) 7085(4) 8572(3) 2492(2) 33(1)

C(14) 8148(4) 10528(3) 3077(2) 35(1)

C(15) 9492(5) 12167(4) 2711(2) 47(1)

C(16) 9774(5) 12122(4) 1746(2) 51(1)

C(17) 7379(5) 10944(4) 1158(2) 40(1)

C(18) 6009(4) 8887(3) 1535(2) 33(1)

C(19) 5545(6) 7159(4) 854(2) 46(1)

C(20) 4313(6) 7288(4) -76(2) 47(1)

C(21) 5555(7) 9354(5) -412(2) 62(1)

C(22) 7816(6) 10647(5) 205(2) 60(1)

C(23) 13112(6) 12004(5) 7193(2) 60(1)

C(24) 9012(7) 10769(5) 7542(2) 59(1)

C(25) 5277(5) 8317(5) 5751(2) 51(1)

C(26) 5301(6) 10819(5) 4032(2) 50(1)

C(27) 9040(5) 7803(5) 2449(2) 45(1)

C(28) 5936(7) 12190(5) 1161(3) 61(1)

C(29) 1721(6) 6885(7) -49(3) 77(1)

C(30) 4364(11) 5693(7) -704(3) 92(2)

O(1) 12374(5) 8216(5) 7547(2) 79(1)

Table S4. Bond lengths [pm] and angles [°] for taraxerone.

____________________________

C(1)-C(2) 153.7(4)

C(1)-C(10) 153.7(4)

C(1)-H(1A) 97.00

C(1)-H(1B) 97.00

C(2)-C(3) 150.2(5)

C(2)-H(2A) 97.00

C(2)-H(2B) 97.00

C(3)-O(1) 119.9(4)

C(3)-C(4) 153.0(4)

C(4)-C(23) 152.2(4)

C(4)-C(24) 154.2(4)

C(4)-C(5) 156.5(4)

C(5)-C(6) 152.3(4)

C(5)-C(10) 155.4(3)

C(5)-H(5) 98.00

C(6)-C(7) 152.7(4)

C(6)-H(6A) 97.00

C(6)-H(6B) 97.00

C(7)-C(8) 154.5(4)

C(7)-H(7A) 97.00

C(7)-H(7B) 97.00

C(8)-C(14) 153.7(4)

C(8)-C(26) 154.5(4)

C(8)-C(9) 156.0(3)

C(9)-C(11) 154.5(4)

C(9)-C(10) 157.1(3)

C(9)-H(9) 98.00

C(10)-C(25) 153.3(4)

C(11)-C(12) 153.0(4)

C(11)-H(11A) 97.00

C(11)-H(11B) 97.00

C(12)-C(13) 153.4(3)

C(12)-H(12A) 97.00

C(12)-H(12B) 97.00

C(13)-C(14) 153.1(3)

C(13)-C(27) 154.2(4)

C(13)-C(18) 157.7(3)

C(14)-C(15) 133.8(4)

C(15)-C(16) 149.4(4)

C(15)-H(15) 93.00

C(16)-C(17) 154.0(4)

C(16)-H(16A) 97.00

C(16)-H(16B) 97.00

C(17)-C(28) 152.0(4)

C(17)-C(22) 153.1(4)

C(17)-C(18) 156.7(3)

C(18)-C(19) 153.5(4)

C(18)-H(18) 98.00

C(19)-C(20) 153.8(4)

C(19)-H(19A) 97.00

C(19)-H(19B) 97.00

C(20)-C(30) 151.3(5)

C(20)-C(29) 152.6(5)

C(20)-C(21) 153.2(4)

C(21)-C(22) 152.5(5)

C(21)-H(21A) 97.00

C(21)-H(21B) 97.00

C(22)-H(22A) 97.00

C(22)-H(22B) 97.00

C(23)-H(23A) 96.00

C(23)-H(23B) 96.00

C(23)-H(23C) 96.00

C(24)-H(24A) 96.00

C(24)-H(24B) 96.00

C(24)-H(24C) 96.00

C(25)-H(25A) 96.00

C(25)-H(25B) 96.00

C(25)-H(25C) 96.00

C(26)-H(26A) 96.00

C(26)-H(26B) 96.00

C(26)-H(26C) 96.00

C(27)-H(27A) 96.00

C(27)-H(27B) 96.00

C(27)-H(27C) 96.00

C(28)-H(28A) 96.00

C(28)-H(28B) 96.00

C(28)-H(28C) 96.00

C(29)-H(29A) 96.00

C(29)-H(29B) 96.00

C(29)-H(29C) 96.00

C(30)-H(30A) 96.00

C(30)-H(30B) 96.00

C(30)-H(30C) 96.00

C(2)-C(1)-C(10) 112.7(2)

C(2)-C(1)-H(1A) 109.0

C(10)-C(1)-H(1A) 109.0

C(2)-C(1)-H(1B) 109.0

C(10)-C(1)-H(1B) 109.0

H(1A)-C(1)-H(1B) 107.8

C(3)-C(2)-C(1) 110.2(3)

C(3)-C(2)-H(2A) 109.6

C(1)-C(2)-H(2A) 109.6

C(3)-C(2)-H(2B) 109.6

C(1)-C(2)-H(2B) 109.6

H(2A)-C(2)-H(2B) 108.1

O(1)-C(3)-C(2) 120.5(3)

O(1)-C(3)-C(4) 122.4(3)

C(2)-C(3)-C(4) 117.1(3)

C(23)-C(4)-C(3) 108.9(3)

C(23)-C(4)-C(24) 107.7(3)

C(3)-C(4)-C(24) 108.4(3)

C(23)-C(4)-C(5) 109.6(2)

C(3)-C(4)-C(5) 107.2(2)

C(24)-C(4)-C(5) 114.8(2)

C(6)-C(5)-C(10) 110.4(2)

C(6)-C(5)-C(4) 113.9(2)

C(10)-C(5)-C(4) 118.1(2)

C(6)-C(5)-H(5) 104.3

C(10)-C(5)-H(5) 104.3

C(4)-C(5)-H(5) 104.3

C(5)-C(6)-C(7) 110.6(2)

C(5)-C(6)-H(6A) 109.5

C(7)-C(6)-H(6A) 109.5

C(5)-C(6)-H(6B) 109.5

C(7)-C(6)-H(6B) 109.5

H(6A)-C(6)-H(6B) 108.1

C(6)-C(7)-C(8) 114.1(2)

C(6)-C(7)-H(7A) 108.7

C(8)-C(7)-H(7A) 108.7

C(6)-C(7)-H(7B) 108.7

C(8)-C(7)-H(7B) 108.7

H(7A)-C(7)-H(7B) 107.6

C(14)-C(8)-C(26) 108.2(2)

C(14)-C(8)-C(7) 110.0(2)

C(26)-C(8)-C(7) 108.3(2)

C(14)-C(8)-C(9) 107.46(19)

C(26)-C(8)-C(9) 114.6(2)

C(7)-C(8)-C(9) 108.2(2)

C(11)-C(9)-C(8) 112.3(2)

C(11)-C(9)-C(10) 113.0(2)

C(8)-C(9)-C(10) 116.73(19)

C(11)-C(9)-H(9) 104.4

C(8)-C(9)-H(9) 104.4

C(10)-C(9)-H(9) 104.4

C(25)-C(10)-C(1) 108.6(2)

C(25)-C(10)-C(5) 114.1(2)

C(1)-C(10)-C(5) 107.9(2)

C(25)-C(10)-C(9) 112.4(2)

C(1)-C(10)-C(9) 107.9(2)

C(5)-C(10)-C(9) 105.80(19)

C(12)-C(11)-C(9) 114.8(2)

C(12)-C(11)-H(11A) 108.6

C(9)-C(11)-H(11A) 108.6

C(12)-C(11)-H(11B) 108.6

C(9)-C(11)-H(11B) 108.6

H(11A)-C(11)-H(11B) 107.6

C(11)-C(12)-C(13) 114.4(2)

C(11)-C(12)-H(12A) 108.7

C(13)-C(12)-H(12A) 108.7

C(11)-C(12)-H(12B) 108.7

C(13)-C(12)-H(12B) 108.7

H(12A)-C(12)-H(12B) 107.6

C(14)-C(13)-C(12) 109.6(2)

C(14)-C(13)-C(27) 108.3(2)

C(12)-C(13)-C(27) 108.6(2)

C(14)-C(13)-C(18) 110.44(19)

C(12)-C(13)-C(18) 107.88(19)

C(27)-C(13)-C(18) 112.0(2)

C(15)-C(14)-C(13) 117.4(2)

C(15)-C(14)-C(8) 122.6(2)

C(13)-C(14)-C(8) 120.0(2)

C(14)-C(15)-C(16) 121.2(2)

C(14)-C(15)-H(15) 119.4

C(16)-C(15)-H(15) 119.4

C(15)-C(16)-C(17) 111.1(2)

C(15)-C(16)-H(16A) 109.4

C(17)-C(16)-H(16A) 109.4

C(15)-C(16)-H(16B) 109.4

C(17)-C(16)-H(16B) 109.4

H(16A)-C(16)-H(16B) 108.0

C(28)-C(17)-C(22) 109.5(3)

C(28)-C(17)-C(16) 107.1(3)

C(22)-C(17)-C(16) 109.0(2)

C(28)-C(17)-C(18) 109.4(2)

C(22)-C(17)-C(18) 110.5(2)

C(16)-C(17)-C(18) 111.3(2)

C(19)-C(18)-C(17) 111.1(2)

C(19)-C(18)-C(13) 112.18(19)

C(17)-C(18)-C(13) 115.8(2)

C(19)-C(18)-H(18) 105.6

C(17)-C(18)-H(18) 105.6

C(13)-C(18)-H(18) 105.6

C(18)-C(19)-C(20) 114.9(2)

C(18)-C(19)-H(19A) 108.5

C(20)-C(19)-H(19A) 108.5

C(18)-C(19)-H(19B) 108.5

C(20)-C(19)-H(19B) 108.5

H(19A)-C(19)-H(19B) 107.5

C(30)-C(20)-C(29) 107.6(3)

C(30)-C(20)-C(21) 110.8(3)

C(29)-C(20)-C(21) 108.9(3)

C(30)-C(20)-C(19) 108.3(3)

C(29)-C(20)-C(19) 110.8(3)

C(21)-C(20)-C(19) 110.5(2)

C(22)-C(21)-C(20) 112.5(3)

C(22)-C(21)-H(21A) 109.1

C(20)-C(21)-H(21A) 109.1

C(22)-C(21)-H(21B) 109.1

C(20)-C(21)-H(21B) 109.1

H(21A)-C(21)-H(21B) 107.8

C(21)-C(22)-C(17) 112.7(3)

C(21)-C(22)-H(22A) 109.0

C(17)-C(22)-H(22A) 109.0

C(21)-C(22)-H(22B) 109.0

C(17)-C(22)-H(22B) 109.0

H(22A)-C(22)-H(22B) 107.8

C(4)-C(23)-H(23A) 109.5

C(4)-C(23)-H(23B) 109.5

H(23A)-C(23)-H(23B) 109.5

C(4)-C(23)-H(23C) 109.5

H(23A)-C(23)-H(23C) 109.5

H(23B)-C(23)-H(23C) 109.5

C(4)-C(24)-H(24A) 109.5

C(4)-C(24)-H(24B) 109.5

H(24A)-C(24)-H(24B) 109.5

C(4)-C(24)-H(24C) 109.5

H(24A)-C(24)-H(24C) 109.5

H(24B)-C(24)-H(24C) 109.5

C(10)-C(25)-H(25A) 109.5

C(10)-C(25)-H(25B) 109.5

H(25A)-C(25)-H(25B) 109.5

C(10)-C(25)-H(25C) 109.5

H(25A)-C(25)-H(25C) 109.5

H(25B)-C(25)-H(25C) 109.5

C(8)-C(26)-H(26A) 109.5

C(8)-C(26)-H(26B) 109.5

H(26A)-C(26)-H(26B) 109.5

C(8)-C(26)-H(26C) 109.5

H(26A)-C(26)-H(26C) 109.5

H(26B)-C(26)-H(26C) 109.5

C(13)-C(27)-H(27A) 109.5

C(13)-C(27)-H(27B) 109.5

H(27A)-C(27)-H(27B) 109.5

C(13)-C(27)-H(27C) 109.5

H(27A)-C(27)-H(27C) 109.5

H(27B)-C(27)-H(27C) 109.5

C(17)-C(28)-H(28A) 109.5

C(17)-C(28)-H(28B) 109.5

H(28A)-C(28)-H(28B) 109.5

C(17)-C(28)-H(28C) 109.5

H(28A)-C(28)-H(28C) 109.5

H(28B)-C(28)-H(28C) 109.5

C(20)-C(29)-H(29A) 109.5

C(20)-C(29)-H(29B) 109.5

H(29A)-C(29)-H(29B) 109.5

C(20)-C(29)-H(29C) 109.5

H(29A)-C(29)-H(29C) 109.5

H(29B)-C(29)-H(29C) 109.5

C(20)-C(30)-H(30A) 109.5

C(20)-C(30)-H(30B) 109.5

H(30A)-C(30)-H(30B) 109.5

C(20)-C(30)-H(30C) 109.5

H(30A)-C(30)-H(30C) 109.5

H(30B)-C(30)-H(30C) 109.5

________________________________

Symmetry transformations used to generate equivalent atoms:

Table S5. Anisotropic displacement parameters (pm^2^x 10^-1^). The anisotropic displacement factor exponent takes the form: -2π^2^[ h^2^a*2U^11^ + ... + 2 h k a* b* U^12^].

________________________________________________________________

U^11^ U^22^ U^33^ U^23^ U^13^ U^12^

________________________________________________________________

C(1) 65(2) 36(1) 38(1) 3(1) 4(1) 16(1)

C(2) 81(2) 41(2) 45(2) 6(1) 2(2) 20(2)

C(3) 63(2) 60(2) 28(1) 4(1) 5(1) 25(2)

C(4) 48(2) 44(2) 35(1) -5(1) 6(1) 13(1)

C(5) 37(1) 35(1) 35(1) -2(1) 6(1) 12(1)

C(6) 57(2) 31(1) 44(2) -7(1) 5(1) 14(1)

C(7) 56(2) 25(1) 42(1) -1(1) 5(1) 9(1)

C(8) 40(1) 26(1) 39(1) 0(1) 5(1) 11(1)

C(9) 39(1) 25(1) 34(1) 2(1) 6(1) 10(1)

C(10) 40(1) 29(1) 36(1) 1(1) 6(1) 9(1)

C(11) 52(2) 26(1) 40(1) 2(1) 2(1) 2(1)

C(12) 41(1) 28(1) 38(1) 1(1) 2(1) 5(1)

C(13) 34(1) 25(1) 35(1) -1(1) 4(1) 9(1)

C(14) 36(1) 27(1) 37(1) 1(1) 2(1) 8(1)

C(15) 51(2) 30(1) 42(2) -2(1) 3(1) -1(1)

C(16) 50(2) 39(2) 44(2) 5(1) 10(1) -5(1)

C(17) 45(1) 29(1) 38(1) 5(1) 8(1) 6(1)

C(18) 33(1) 27(1) 36(1) 2(1) 5(1) 10(1)

C(19) 62(2) 35(1) 39(1) -2(1) -3(1) 19(1)

C(20) 59(2) 40(2) 37(1) 1(1) 0(1) 18(1)

C(21) 79(2) 51(2) 37(1) 8(1) 7(1) 7(2)

C(22) 65(2) 49(2) 44(2) 2(1) 19(2) -3(2)

C(23) 58(2) 62(2) 45(2) -8(1) -4(1) 12(2)

C(24) 70(2) 65(2) 43(2) -5(1) 13(2) 26(2)

C(25) 46(2) 55(2) 44(2) 4(1) 12(1) 12(1)

C(26) 56(2) 54(2) 50(2) 1(1) 7(1) 33(1)

C(27) 48(2) 48(2) 43(1) -3(1) 2(1) 25(1)

C(28) 77(2) 36(2) 72(2) 12(1) 8(2) 25(2)

C(29) 54(2) 92(3) 58(2) 15(2) -5(2) 8(2)

C(30) 152(5) 86(3) 48(2) -21(2) -20(2) 71(3)

O(1) 85(2) 86(2) 65(2) 13(1) -15(1) 41(2)

_______________________________________________________________
